# How Ionic Strength Affects the Conformational Behavior of Human and Rat Beta Amyloids – A Computational Study

**DOI:** 10.1371/journal.pone.0062914

**Published:** 2013-05-23

**Authors:** Zdeněk Kříž, Jiří Klusák, Zdena Krištofíková, Jaroslav Koča

**Affiliations:** 1 Central European Institute of Technology (CEITEC), Masaryk University, Brno, Czech Republic; 2 National Centre for Biomolecular Research, Faculty of Science, Masaryk University, Brno, Czech Republic; 3 Alzheimer Disease Centre, Prague Psychiatric Centre, Prague, Czech Republic; University of Akron, United States of America

## Abstract

Progressive cerebral deposition of amyloid beta occurs in Alzheimeŕs disease and during the aging of certain mammals (human, monkey, dog, bear, cow, cat) but not others (rat, mouse). It is possibly due to different amino acid sequences at positions 5, 10 and 13. To address this issue, we performed series of 100 ns long trajectories (each trajectory was run twice with different initial velocity distribution) on amyloid beta (1–42) with the human and rat amino acid sequence in three different environments: water with only counter ions, water with NaCl at a concentration of 0.15 M as a model of intracellular Na^+^ concentration at steady state, and water with NaCl at a concentration of 0.30 M as a model of intracellular Na^+^ concentration under stimulated conditions. We analyzed secondary structure stability, internal hydrogen bonds, and residual fluctuation. It was observed that the change in ionic strength affects the stability of internal hydrogen bonds. Increasing the ionic strength increases atomic fluctuation in the hydrophobic core of the human amyloid, and decreases the atomic fluctuation in the case of rat amyloid. The secondary structure analyses show a stable α-helix part between residues 10 and 20. However, C-terminus of investigated amyloids is much more flexible showing no stable secondary structure elements. Increasing ionic strength of the solvent leads to decreasing stability of the secondary structural elements. The difference in conformational behavior of the three amino acids at position 5, 10 and 13 for human and rat amyloids significantly changes the conformational behavior of the whole peptide.

## Introduction

Alzheimer's disease (AD) is a neurodegenerative disorder that affects more than 30 million people worldwide. AD patients experience a progressive decline in their cognitive functions and loss of short term memory with aging [1]. The brain regions most commonly affected in AD are the hippocampus and entorhinal cortex, both of which are involved in short-term memory and learning processes [2]. Histopathological examination of AD brain slices reveals amyloid-beta peptide (Aβ) deposits. The extracellular plaques mainly contain aggregates of amyloid-beta (Aβ) peptides. However, more recent research suggests that soluble monomeric or oligomeric amyloid is first deposited in the neuron and later in the extra-cellular space and that this intra-cellular accumulation of amyloid is one of the first neurodegenerative alterations in AD brains [3].

**Figure 1:**
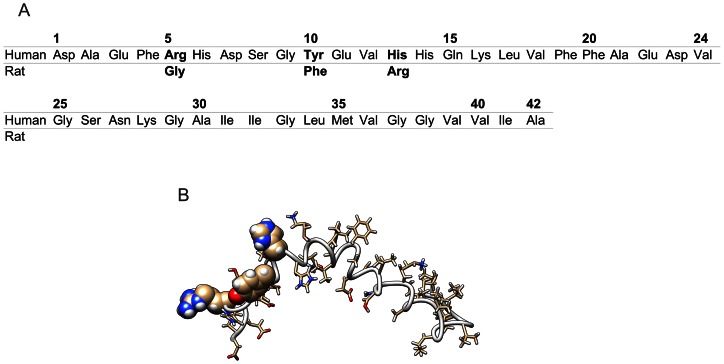
Amino acids sequence of human and rat amyloid β with highlighted residues which are different (A). Starting structure used for MD simulations with highlighted residues mutated in rat amyloid (B).

Aβs are the products of the γ-secretase cleavage of a large membrane-spanning glycoprotein, the amyloid precursor protein (APP). The cleavage by γ-secretase is not residue sequence specific and Aβ peptides of lengths between 39 to 43 amino acids are formed. The most abundant peptides are Aβ(1–40) and Aβ(1–42) in a ratio of about 10: 1. The Aβ(1–42) is significantly more neurotoxic and is also the major constituent of senile plaques. The sequence of Aβ(1–42) together with used starting structure is showed on Figure 1.

The first 16 residues of Aβ are largely hydrophilic. The remaining residues comprise a largely hydrophobic domain. The residues HIS13 and HIS14 form a binding site for Cu(II) [4]. The central region, Aβ(12–23), has been identified as the self-recognition site [5], [6] for the formation of dimers and higher oligomers. This region is also the binding site for cholesterol [7], apolipoprotein E (apoE) [8], α7nAChR [9], and amyloid beta-peptide binding alcohol dehydrogenase [10]. Aβ also binds with high affinity to the enzyme, catalase [11], but in the region Aβ(31–35) [12].

The solution structure of monomeric Aβ in water is still unknown. Two NMR structures of Aβ(1–40), determined in SDS micelles [13] and 40% trifluoroethanol [14], exhibited extensive α-helical content, while a CD analysis of aqueous Aβ(1–40) gave a mixture of coils, turns, β-sheets and α-helices, and a high β-sheet content at air–water [15] or membrane interfaces [16]. An NMR structure determination of aqueous Aβ(10–35), revealed a stable “collapsed coil” core structure, reported to be the same as that of Aβ(1–40), without α-helical or β-sheet secondary structure [4].

It is now known that the major toxicity of Aβ is neither from monomers nor from insoluble fibrils. The most toxic species are soluble oligomeric structures of Aβs ranging from small oligomers to protofibrils. Detailed structures of Aβ oligomers are not revealed. High resolution atomic force microscopy images of soluble Aβ(1–42) monomers and oligomers deposited on a mica surface confirmed the hairpin fold of monomers and ordered structure of small oligomers [17]. On the other hand, scanning tunneling microscopy of Aβ(1–40) monomers and oligomers deposited on a gold surface gave different results. For monomeric Aβ, three or four parallel folds were found and oligomers were identified as assembles of such folded monomers connected end-to-end [18]. In all cases, the monomeric peptide must undergo conformational changes involving a fold near GLY25 and form a β-sheet structure before aggregation.

It is known that the stability of peptides is also affected by salts. Monovalent salts, such as sodium chloride, often affect protein stability by modifying the ionic strength of the solution, which can be overall slightly stabilizing or destabilizing, depending on the nature of the specific charge distribution within the protein. Coulombic interactions at the protein surface are efficiently screened in the presence of salt. Their contributions to the thermodynamic stability of a protein can thus be determined experimentally by measuring unfolding transitions as a function of salt concentration. However, interpreting the results from such studies is not always straightforward given the complex nature of salt effects. In some cases, ions stabilize proteins by high-affinity binding to specific sites. This ligand-induced stabilization is ion-specific and usually observed in the range of 0–0.2 M salt. Bulk ionic strength effects play a role in screening surface charge–charge interactions [19]. High concentrations of inorganic salts, such as NaCl, favor compact protein conformations because they are excluded from the protein surface [20].

Experimental studies performed on Aβ(1–42) and Aβ(1–40) using atomic force microscopy (AFM) and Western blot analysis by Stine *et. al.* [21] showed that the oligomerization of Aβ(1–40) is much slower than that of Aβ(1–42) (6 weeks versus 24 hours). These authors tried various combinations of pH and ionic strength and detected fibril formation at acidic pH and low ionic strength. Increasing the ionic strength while maintaining an acidic pH resulted in the formation of dense fibrilar aggregates. At neutral pH and low ionic strength, oligomers with the addition of several short fibrils were found. Increasing the ionic strength led the fibrils to coalesce into primarily small oligomeric aggregates. Oligomer formation was favored at neutral pH and physiological ionic strength.

Aβ also forms age-related fibrils and deposits in the brains of various mammals, including bears, cows, dogs, cats and all non-human primates that have been analyzed. The amino acid sequence of Aβ in these animals is identical to that of humans. No Aβ deposits have yet been found in the brains of normal aged rats and mice. It suggests that the three amino acid differences between their Aβ and that of amyloid-bearing mammals may impede the fibrillogenicity of Aβ [22], [23]. This is the reason why special transgenic mice are used as a model of Alzheimeŕs disease. In this study we performed a series of molecular dynamics simulations in an explicit solvent model with different concentrations of NaCl in order to understand the differences in the conformational behavior of both amyloids at various ionic strengths of solvent.

## Methods

The starting structure of the human amyloid for molecular dynamics simulations was taken from the Brookhaven Protein Database. The first structure from the ensemble of 30 structures solved by NMR with pdb code 1Z0Q [24] was used. The structure of the rat amyloid was prepared using *in silico* mutations from the human amyloid where the followed residues were changed: ARG5 to GLY5, TYR10 to PHE10 and HIS13 to ARG13. It was performed using Triton [25] interfaced with Modeller software [26]. All structures were solvated with a 12 Å thick water shell represented by the TIP3P water model [27]. This was done with the Solvate [28] software. In the first case, only counter ions for neutralizing the charge were used. In order to mimic physiological conditions, NaCl at a concentration of 0.15 M was added using Solvate as a model of intracellular conditions at steady state. Increasing the NaCl concentration to 0.30 M as a model of intracellular conditions in the stimulated state was done using the Leap program of the Amber software package [29]. The Amber version 11 software package together with AmberTools version 1.2 were used for simulations and trajectory analyses. An octahedral simulation box was added to the system using Leap program to allow the simulation with periodic boundary conditions. The Duan *et. al.* all atom force field *ff03* [30] was used for all simulations. The force field and water model were chosen based on a force fields comparison by Florová *et. al.* [31] and Hornak *et. al.* [32]. The systems were relaxed and slowly heated to simulation temperature before the production phase of the MD simulations. The first step was the minimization and MD simulation of the solvent molecules and ions. The low-temperature (at 5 and 10 K) MD simulations of the side chains, solvent molecules and ions with decreasing restrain forces applied to backbone atoms were followed by low-temperature (at 5 and 10 K) MD simulations of the whole systems. In the final steps of the relaxation procedure, the simulation systems were slowly heated to 298.16 K for 200 ps followed by a 50 ps long MD simulation under NPT conditions. For a detailed description of the MD protein equilibration protocol, see [33]–[35]. The Sander program [29] of the Amber software package was used for the equilibration phase of the simulations.

The production phase of MD was 100 ns long for each system and was run twice with different velocity distribution using different random seed. The Pmemd program from the Amber software package was used for the simulations. The particle-mesh Ewald (PME) method was used for dealing with electrostatic interactions, and all simulations were performed under periodic boundary conditions in the [NpT] ensemble at 298.16 K and 1 atm using a 2 fs integration step. The SHAKE algorithm [36], with a tolerance of 10^−5^ Å, was used to fix the positions of all hydrogen atoms, and a 9.0 Å cutoff was applied to non-bonding interactions. A Berendsen thermostat was used [37].

The potential and kinetic energy together with the density of the systems were monitored during the production phases. The evolution of the trajectories was monitored as changes in the values of the radius of gyration and RMS deviation calculated using the Ptraj module of the Amber software package. The RMSD values were calculated for all atoms in the molecule with atomic weight included into calculation.

Secondary structure elements were monitored using the Ptraj module. The secondary structure classification is based on the Kabsch and Sander DSSP program [38] where the classification is based on hydrogen bond patterns in combination with backbone torsion values. The values of φ and ψ backbone torsions were also monitored during MD simulations and time-dependent Ramachandran plots for all residues were compiled. The intramolecular hydrogen bonds were investigated and their stability was analyzed. The local flexibility of investigated molecules were monitored using a calculation of atomic and residual fluctuations. This was performed by the Ptraj module of the Amber program package. The population and evolution of amyloid conformations was assessed using the K-means clustering algorithm implemented in the Ptraj module. The RMSDs of protein backbone atoms were used for clustering and the number of clusters were iteratively set to 20 by following indices measuring clustering performance, as described by Shao *et al.* [39].

## Results and Discussion

### Stability of the simulated trajectories

**Figure 2:**
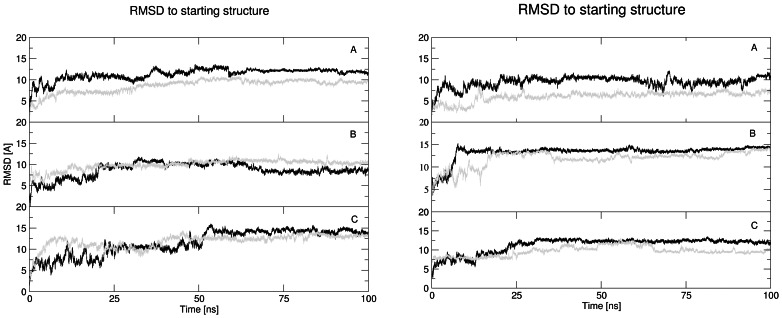
Root mean square deviation of amyloid backbone during molecular dynamics simulations on amyloid with human (left) and with rat (right) amino acid sequence. Graphs A, B, C represent simulations with NaCl concentrations of 0.00, 0.15, and 0.30 M, respectively. The first MD run is represented by black color and the second MD run by grey color.

The RMSD to the starting structure calculated for all twelve trajectories are summarized in Figure 2. Large conformational changes are seen during the first 25 ns of the simulations with an increase in the RMSD of 10 to 12 Å. The amyloid with the human amino acid sequence and with a NaCl concentration of 0.15 M exhibits, after initial conformational changes, a relatively stable conformational evolution with a RMSD value of around 10 Å. The reason for the large RMSD to the experimental structure is probably the fact that the experimental data was obtained in a mixture of hexafluoroisopropanol with water, and our simulations were only performed in pure water. The simulations with an NaCl concentration of 0.30 M gave additional conformational changes with RMSD values of around 14 Å after 55 ns. We have performed analysis of RMSD and end-to-end distances for all 30 structures included in pdb file 1Z0Q. The RMSD values calculated in the same way as in the trajectories gave the values between 0.0 and 10.3 Å. The end-to-end distances differs between 5.8 and 53.0 Å. It means that our results are in agreement with experimentally obtained data.

As in the human Aβ case, simulations of amyloid with rat amino acids sequence also indicated the most stable trajectories with a NaCl concentration of 0.15 M and 0.30 M, with an RMSD value of 14 Å or 12.5 Å, respectively. The simulations with NaCl concentration of 0.00 M showed convergence to two different conformations in both human and rat cases. The differences between both conformations are only in orientation of N- and C-ends of molecules.

**Figure 3:**
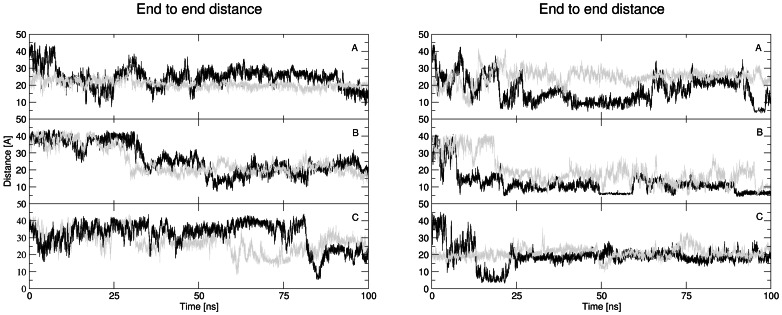
Calculated end to end distance values during molecular dynamics simulations of amyloid with human (left) and with rat (right) amino acid sequence. Graphs A, B, C represent simulations with NaCl concentrations of 0.00, 0.15, and 0.30 M. The first MD run is represented by black color and the second MD run by grey color.

The globular structures were indicated by monitoring the end to end distance during simulations (see Figure 3). With the human amyloid, all simulations exhibited similar behavior with a starting distance around 40 Å decreasing to 20 Å during the simulations.

The simulations on Aβ with the rat amino acid sequence indicated hydrogen bonds between both ends of the peptide, which were very stable in the simulation with 0.15 M NaCl concentration in the second half of simulations. Similar behavior, but with less stable hydrogen bonds, was seen in the simulation with 0.30 M NaCl in first quater of trajectories and also simulation with 0.00 M NaCl at the end of simulation.

The calculated radius of gyration values during the trajectory (summarized in Figure S1) indicate a more globular structure than the starting structures. In all cases, the radius of gyration values change for the first 10 ns and are then stable for the rest of the simulations.

### Residual fluctuation analyses

**Figure 4:**
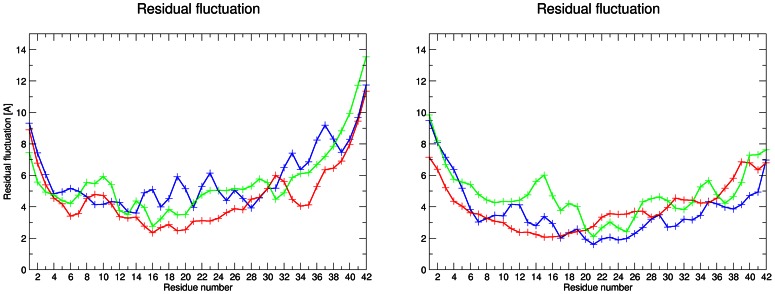
Calculated residual fluctuations for amyloid with human (left) and rat (right) amino acid sequence. The simulations are colored as follows: c(NaCl) 0.00 M – blue, 0.15 M – red and 0.30 M – green.

Calculated residual fluctuations (see Figure 4) indicate some, although no significant, influence of ionic strength on residual flexibility. It is seen that the lowest fluctuations are located in the hydrophobic core of the amyloids. The highest fluctuations are found at terminal residues as expected. In the amyloid with the human amino acid sequence, the C-terminal residues exhibited a higher fluctuation than the N-terminal residues. Residues ASP7-GLU11 exhibited a higher fluctuation than the neighboring residues for simulations with 0.15 and 0.30 M NaCl. Even if the lowest fluctuation values are found for physiological steady-state conditions, simulations with the other NaCl concentrations exhibit similar behavior.

The largest differences between the residual fluctuations of the rat and human amyloids are in the mutated residues region. The peak located between residues ASP7 and GLU11 in the human amyloid is not present in the rat amyloid. However, in the simulation with 0.30 M NaCl, residues GLU11 to LEU17 of the rat amyloid exhibited a higher flexibility than the neighboring residues. In this case, the change in ionic strength from physiological steady-state conditions leads to a decrease in ALA21 to SER26 flexibility. The C-end of the rat amyloid exhibited lower atomic fluctuations in the rat Aβ than in the human Aβ for each ionic strength tested.

### Intramolecular H-bond analyses

Statistical data for H-bond analyses are summarized in the Table 1. For the human amyloid, analyses of intramolecular hydrogen bonds (defined by the distance between donor and acceptor being less than or equal to 3.5 Å and the angle between acceptor, donor and hydrogen less than 30 degrees), performed on molecular dynamics trajectories. The simulations with NaCl concentration of 0.15 M showed the most stable hydrogen bonds (8 with ocupancy between 80 and 100%) in comparison with simulations with other NaCl concentrations. Most of all stable H bonds with high occupancy are presented inside of hydrophobic core of amyloid. A hydrogen bond between PHE4 and ASP7 or SER8 was observed in the simulation with 0.30 M NaCl. This H-bond constantly switches from being between ALA2 and ARG5 to between GLU3 and SER8 and vice versa. Stable H-bonds were mainly observed between the backbone parts of amino acids. Only SER26 or GLN15 side chains were involved in H-bond stabilization and these H-bonds were broken in the highest ionic strength simulation. Another H-bond between ILE31 and LEU34 was observed in simulations with the lowest and highest ionic strength simulations. This H-bond was broken in the simulation with 0.15 M NaCl.

**Table 1:** Number of intramolecular H-bonds with calculated occupancies found during the MD simulations.

Human amyloid-beta			
H-bond occupancy	c(NaCl) = 0.00 M	c(NaCl) = 0.15 M	c(NaCl) = 0.30 M
80–100%	6 (5)	8 (6)	5 (5)
60–80%	6 (4)	3(2)	7(3)
40–60%	5 (2)	9 (1)	10(2)
20–40%	21 (3)	13 (1)	14 (4)
**Rat amyloid-beta**			
80–100%	11 (5)	7 (5)	5 (3)
60–80%	9 (3)	8 (4)	10 (4)
40–60%	8 (1)	8 (1)	7 (0)
20–40%	15 (2)	19 (2)	21 (7)

Numbers in parentheses indicate number of H-bonds in the stable helical region between residues PHE10/TYR10 and ALA21. The hydrogen bond is defined as donor-acceptor distance being less than or equal to 3.5 Å and acceptor-donor-hydrogen angle being less than 30 degrees.

In the rat amyloid simulations with higher variances for different ionic strengths (13 intramolecular H-bonds for the simulation in pure water with occupancy large than 80%, 8 H-bonds for the simulation with 0.15 M NaCl and only 5 H-bonds in the simulation with 0.30 M NaCl).

Moreover, the simulation in pure water shows another stabilization between the residues at the C-end of the amyloid (ILE32– Val36, ILE31– LEU34 and ALA30– GLY33). These H-bonds were broken at simulations with higher ionic strength. A relatively stable H-bond between ILE41 and HIS6 backbone was seen in the simulation with 0.15 M NaCl. Stabilization of the structure between the backbone oxygen of the ALA42 and the side chain of ARG13 with an occupancy of around 30% was also observed in all simulations. The replacement of HIS13 with ARG13 led to the creation of a large number of intramolecular H-bonds, not only with the C-end of the molecule, but also with the side chains of GLU3 or ASP7. On the other hand, replacing ARG5 with GLY5 led to the loss of the possibility of stabilizing the structure due to the H-bond created by the relatively flexible side chain observed in human amyloid simulations with an occupancy between 30 and 40%. Detailed information on the H-bonds with distances, angles between acceptor-donor-hydrogen and occupancies are summarized in Tables S1–S6 in the Supplementary material.

### Secondary structure analyses

**Figure 5:**
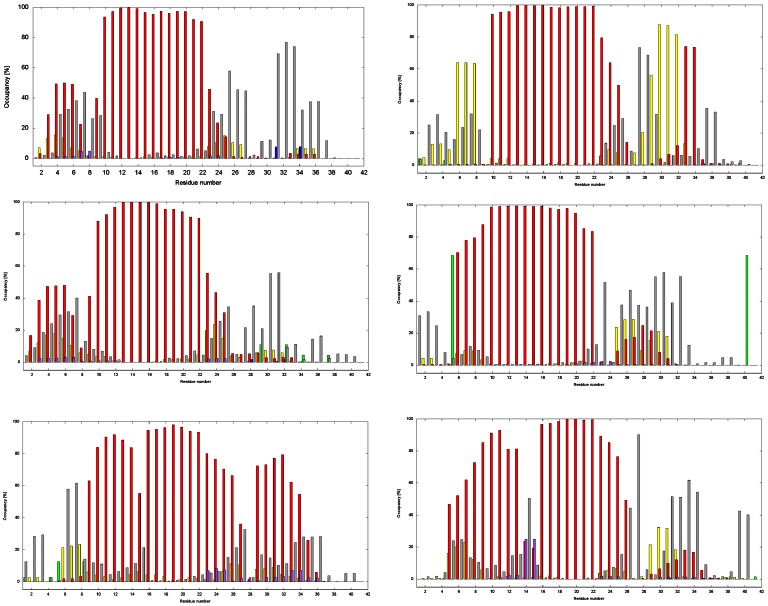
Histograms of frequency occurrence of secondary structure elements calculated from MD trajectories for amyloids with human (left) and with rat (right) amino acid sequence for different concentrations of NaCl (upper –0.00 M, middle –0.15 Mand lower –0.30 M). Secondary structures are represented by the following colors: 3_10_-helix – yellow, α-helix – red, π-helix – violet, parallel β-sheet – blue, anti-parallel β-sheet – green and turns – silver.

In the amyloid with the human amino acid sequence, secondary structure analyses (see Figure 5) gave a stable hydrophobic helical core in all simulations. This region is located between residues TYR10 and GLU22. Simulations with NaCl concentration of 0.00 and 0.15 M showed another short helix between residues PHE4 and HIS6. The stability of this small region is lower than that of hydrophobic core region. The higher ionic strength led to destabilization of this short region with higher population of γ-turn secondary structure elements. Higher ionic strength also destabilizes hydrophobic core regions. The largest change in secondary structure stability was determined for residue GLN15. In this case the second relatively stable region was found between residues GLY29 and LEU34. The β-sheet secondary structure element was found only with very low stability for residues SER8, ILE31 and LEU34 in simulations with low ionic strength.

For the amyloid with the rat amino acid sequence, the stable α-helix core was broader than in the human amyloid. This region was located between residues PHE10 and VAL24 for simulations with 0.00 M NaCl and between residues PHE10 and ALA21 under physiological steady-state conditions. This region was destabilized in simulation with NaCl concentration 0.30 M between residues HIS14 and GLN15. At the N-terminus, a second short 3_10_-helix structure was observed between residues ARG5 and SER8 and at the C-terminus between residues LYS28 and ILE32 for NaCl concentrations of 0.00 M. In this case, a stable α-helix was formed between residues GLY33 and LEU34. This helix was broken at a NaCl concentration of 0.15 and 0.30 M. Under physiological steady-state conditions, an antiparallel β-sheet secondary structure for residues HIS5 and VAL40 was observed.

### Ramachandran plot

**Figure 6:**
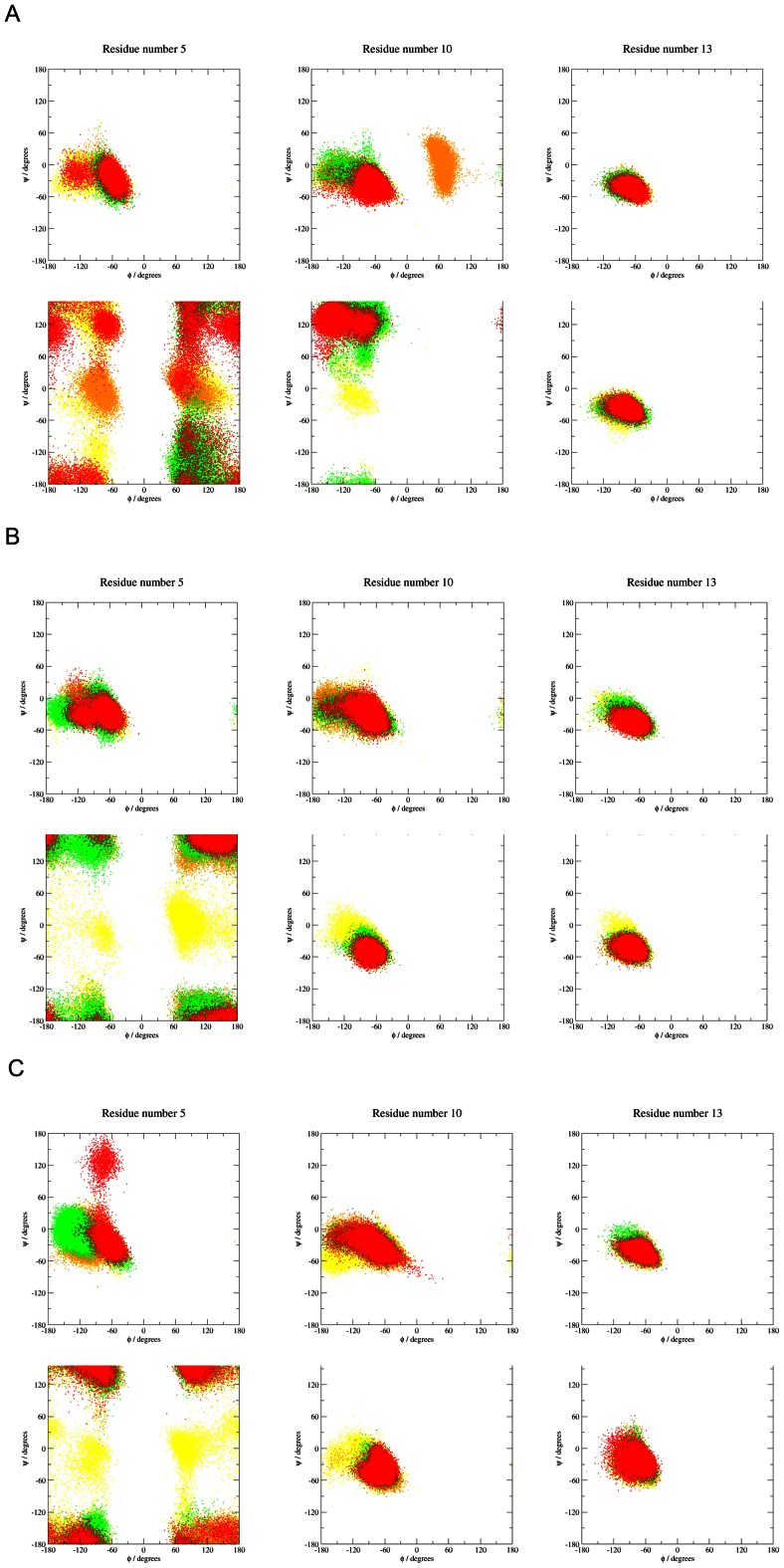
Time-dependent Ramachandran diagrams for residues 5, 10 and 13 which are different for amyloids with human (upper) and rat amino acids sequence (lower). A) Analyses for 0.0 M, B) for 0.15 M and C) for 0.30 M NaCl concentration. First quarter of trajectory is depicted in yellow, the next quarter is in orange followed by green and the last part of the trajectories is depicted in red.

The behavior of the φ and ψ backbone torsions of the three different residues of the human and rat amyloids is depicted in Figure 6. With the human amyloid peptide, the bulky ARG at the fifth position exhibited almost the same behavior in all simulations, occupying the α-helix region of the Ramachandran diagram. On the other hand, the conformational behavior of TYR10 was different in the simulation with zero ionic strength compared to the other two simulations. The beginning of all the simulations was almost the same. However, with zero ionic strength, a rotation of the φ torsion from values between −180 and −50 degrees to values of around 60 degrees at the end of the simulations was observed. These differences in conformational behavior were also reflected by the neighboring GLY9. In this case, the simulations with NaCl concentrations of 0.00 and 0.30 M exhibited relatively large stabilization in the final quarter of simulations compared to the simulation with an NaCl concentration of 0.15 M. The conformational behavior of the HIS13 backbone in all simulations was almost the same as that of ARG5.

As expected, with the rat amyloid, the replacement of ARG with the small GLY at the fifth position changed the flexibility of this part of the peptide. The conformational behavior of the GLY5 mutant was very similar in all simulations. On the other hand, changing TYR to PHE at the tenth position led to different behavior in all the simulations. With zero ionic strength, the Ramachandran plot was located in the β region, but it was in the helical regions for the other simulations. The replacement of HIS with ARG at position 13 didn't affect the conformational behavior of the backbone part of the amino acid. Complete time-dependent Ramachandran plots for all residues and all simulations are given in the Figures S2–S7 for human amyloid and Figures S8–S13 for rat amyloid.

### Clustering of conformations

The RMS values calculated for backbone atoms were used for snapshots of the clustering found in molecular dynamics simulations. A K-means cluster analysis with K = 20 was used. The characteristics of the clusters, such as conformation occurrence in the cluster together with calculated hydrophilic and hydrophobic parts of solvent-accessible surface area are summarized in the Table 2 for human amyloid and in the Table 3 for rat amyloid, respectively. The representative structures of all obtained clusters are saved in pdb format in the supplementary material (Cluster S1).

**Table 2:** Occurrence of conformations in clusters in % found by K-means algorithm with K = 20 for all trajectories of amyloid with human amino acid sequence.

Cluster number	c(NaCl) = 0.00 M			c(NaCl) = 0.15 M			c(NaCl) = 0.30 M		
	Occurrence [%]	Hydrophobic SASA [Å^2^]	Hydrophilic SASA [Å^2^]	Occurrence [%]	Hydrophobic SASA [Å^2^]	Hydrophilic SASA [Å^2^]	Occurrence [%]	Hydrophobic SASA [Å^2^]	Hydrophilic SASA [Å^2^]
1	0.9	2134.7	2381.9	1.5	1850.6	2339.2	2.0	2073.8	2423.7
2	23.9	1726.5	1925.1	9.0	1715.4	2105.9	5.1	1387.7	2172.4
3	1.0	2092.9	2366.1	5.1	1970.6	2028.9	2.5	1695.4	2208.9
4	0.4	2075.1	2294.9	3.1	1560.3	2276.3	1.0	1604.6	2299.4
5	17.0	1734.9	2072.2	1.2	2124.9	2442.5	1.1	2041.1	2265.7
6	0.5	2066.7	2528.9	8.3	1680.5	2104.4	4.5	1593.5	2204.4
7	1.0	2130.5	2367.4	1.9	1951.4	2241.1	2.6	1504.8	2222.9
8	6.9	1623.2	2333.0	2.3	1934.4	2245.2	3.8	1755.7	2170.8
9	0.3	2006.0	2479.4	18.2	1759.5	2272.2	6.8	1726.8	2097.8
10	1.9	1666.9	2291.1	2.2	1647.0	2224.5	1.7	1981.7	2294.0
11	23.5	1589.4	2102.5	8.0	1759.8	2124.2	1.5	2002.4	2294.1
12	0.3	2069.7	2558.6	5.6	2045.9	2081.4	8.1	1657.3	2408.8
13	4.1	1682.7	2412.7	1.5	1887.6	2180.9	1.4	1903.0	2168.1
14	0.3	2244.9	2430.6	1.9	1691.9	2167.2	5.3	1800.9	2206.3
15	10.6	1660.7	2143.0	1.0	2053.7	2343.5	5.1	1466.1	2254.3
16	2.5	1622.8	2109.9	1.2	1940.4	2256.7	26.1	1592.3	2226.9
17	0.6	1911.9	2334.9	7.2	1887.9	2064.0	7.7	1677.9	2378.5
18	3.1	1595.2	2312.4	11.1	1622.0	2241.5	10.8	1485.3	2264.7
19	0.6	2344.6	2447.8	0.9	1976.7	2218.9	1.0	2010.9	2331.1
20	0.6	2274.3	2361.9	8.9	1859.1	2185.9	1.9	2169.3	2225.9

Hydrophobic and hydrophilic parts of solvent accessible surface area [in Å^2^] calculated for representative structures.

**Table 3:** Occurrence of conformations in clusters in % found by K-means algorithm with K = 20 for all trajectories of amyloid with rat amino acid sequence.

Cluster number	c(NaCl) = 0.00 M			c(NaCl) = 0.15 M			c(NaCl) = 0.30 M		
	Occurrence [%]	Hydrophobic SASA [Å^2^]	Hydrophilic SASA [Å^2^]	Occurrence [%]	Hydrophobic SASA [Å^2^]	Hydrophilic SASA [Å^2^]	Occurrence [%]	Hydrophobic SASA [Å^2^]	Hydrophilic SASA [Å^2^]
1	1.2	2019.9	2048.0	0.1	2146.1	2316.1	1.8	2056.9	1985.8
2	1.7	1761.5	2005.6	41.3	1608.2	1921.4	29.5	1396.7	2133.3
3	14.2	1765.4	1820.1	0.6	2173.3	2212.9	4.2	1607.8	1915.3
4	6.8	1780.0	1888.7	0.3	2218.3	2115.7	0.7	2087.7	2143.1
5	3.9	1620.1	1940.8	6.8	1748.0	2045.3	1.3	2068.2	2069.6
6	2.5	1891.7	2007.5	0.9	2096.2	2191.6	2.0	1539.8	1976.5
7	2.0	1730.3	2032.4	2.6	1677.2	1849.6	2.5	1609.6	2098.1
8	1.0	1502.8	2030.7	0.7	2357.6	2209.5	0.6	2079.6	2138.1
9	1.1	2047.7	2050.9	0.2	2174.9	1979.4	0.8	2025.3	2053.0
10	11.6	1743.7	1970.6	1.5	1680.1	2049.8	1.3	1812.0	1872.3
11	4.9	1631.1	1878.7	0.6	2205.2	2132.2	2.2	1507.4	1975.7
12	5.6	1708.7	2068.7	0.6	2260.2	2177.1	10.5	1330.1	2161.3
13	3.8	1819.2	1959.9	0.4	2083.5	2210.3	2.5	1997.2	1969.8
14	2.6	1956.8	2013.5	9.1	1653.2	1936.6	0.6	2175.7	2014.8
15	0.5	1890.2	2070.9	0.9	2243.0	2124.5	29.9	1438.9	1992.6
16	3.6	1837.9	2234.2	0.5	2095.1	2246.1	2.1	2122.6	2141.5
17	3.4	1861.6	1997.8	0.7	2089.3	2242.1	3.1	1752.2	1934.7
18	24.7	1819.1	1816.7	9.9	1553.8	1807.0	0.9	1869.5	2003.1
19	2.4	1949.9	2016.2	1.0	2064.1	2165.8	1.3	2237.4	2160.4
20	2.6	1854.4	2198.0	21.5	1593.2	1805.0	2.0	1761.4	2061.2

Hydrophobic and hydrophilic parts of solvent accessible surface area [in Å^2^] calculated for representative structures.

With the human amyloid, the simulation at 0.00 M NaCl gave two large clusters with a populations of 23.9% and and 23.5%. Other two clusters with populations of 17 and 10.6%, and 3 clusters with populations between 3.0 and 10.0%.

Hydrophobicity is predicted to be one of the driving processes in fibril formation [40] therefore the hydrophobic and hydrophylic parts of solvent-accessible surface area (SASA) were calculated. In this case, the most populated cluster also exhibited the smallest values for hydrophobic (1726.5 Å^2^) and hydrophilic (1925.1 Å^2^) parts of solvent-accessible surface area (SASA).

The most populated clusters in the simulation with 0.15 M NaCl had an occurrence of only 18.2% or 11.1%, respectively. In this simulation 7 clusters with occupancy between 3.0 and 10.0% were found. Other clusters exhibited occupancy less than 3.0%. In this case, the most populated cluster did not exhibit the smallest hydrophobic and hydrophilic parts of the SASA, which was found for the cluster with a population of 8.3%.

The distribution of conformational cluster populations for the simulation with 0.30 M NaCl was different from the other two simulations. Only one cluster was found with a population of 26.1% and one cluster with population of 10.8%. It was found also 8 clusters with population between 3.0 and 10.0%. Other 10 clusters exhibited populations less than 3.0%. In this case the most populated clusters not exhibited the smallest values of both hydrophobic and hydrophilics parts of SASA. The smallest value of hydrophobic part of SASA was found for cluster with population of 5.1%.

The distribution of conformers in the clusters for simulations of rat amyloid is slightly different from that with the human amyloid. In the simulation with zero ionic strength, three clusters with a population larger than 10% were found. The most populated cluster had a population of 24.7%. Only one cluster with a population less than 1.0% was found. The distribution of clusters with populations between 1.0 and 10% was as follows: 2 clusters with a population larger than or equal to 5% and 14 clusters with a population less than 5%. In this case, all clusters showed the ratio between hydrophobic and hydrophiclic parts of SASA larger than 0.8 and the most populated cluster exhibited ratio 1.0. The values obtained were 1819.1 Å^2^ for the hydrophobic and 1816.7 Å^2^ for the hydrophilic parts of the SASA.

In contrast, the simulation of the rat amyloid with a NaCl concentration of 0.15 M gave two large clusters with a population of 41.3% and 21.5%. Three clusters with a population between 3.0 and 10.0% were found. Also in this simulation, the most populated cluster exhibited the smallest values for the hydrophobic and hydrophilic parts of its SASA with values of 1608.2 Å^2^ for the hydrophobic and 1921.4 Å^2^ for the hydrophilic parts.

In the simulation with 0.30 M NaCl, two largest clusters had a population of 29.9% and 29.5%. The distribution of the remaining clusters was: one cluster with a population of 10.5% and 5 clusters with a population of less than 1.0%. In this case the most populate cluster also exhibited the best ratio between the hydrophobic and hydrophiclic parts of its SASA with values of 1438.9 Å^2^ for the hydrophobic and 1992.6 Å^2^ for the hydrophilic part of the SASA, respectively.

Statistical analysis of SASA hydrophobic vs. hydrophilic values calculated for MD simulation snapshots (summarized in the Table 4) indicate decreasing hydrophilic part of SASA in the rat amyloid compared to the human amyloid, which is due to changes in the amino acids at the 5^th^, 10^th^ and 13^th^ positions. Human amyloid exibited the smallest average hydrophobic parts of SASA for simulation with 0.00 M NaCl, but values for simulation with 0.30 M NaCl are similar. On the other hand, rat amyloid exhibited the smallest average hydrophobic parts of SASA in simulations with 0.30 M NaCl and largest values in simulation with 0.00 M NaCl. On the other hand, the values for the hydrophilic part of the SASA were similar in all simulations, with smaller values for the rat amyloid.

**Table 4:** Statistical data of hydrophobic and hydrophilic parts of solvent accessible surface area (SASA) calculated from MD simulations.

c(NaCl) [M]	Hydrophobic part of SASA [Å^2^]			Hydrophilic part of SASA [Å^2^]		
	Minimum	Maximum	Average	Minimum	Maximum	Average
**Human amyloid**						
0.00	1241.1	2434.2	1674.3±153.9	1747.2	2774.0	2181.8±152.5
0.15	1455.8	2277.0	1842.7±125.4	1866.7	2629.9	2202.9±99.7
0.30	1239.5	2374.8	1687.4±182.8	1858.3	2707.4	2217.8±116.8
**Rat amyloid**						
0.00	1300.2	2252.9	1756.7±119.2	1660.6	2369.4	1982.8±99.5
0.15	1325.8	2491.7	1652.6±161.8	1597.9	2467.2	1899.2±119.6
0.30	1164.5	(2427.0	1550.2±234.1	1746.7	2382.6	2052.3±86.0

**Figure 7:**
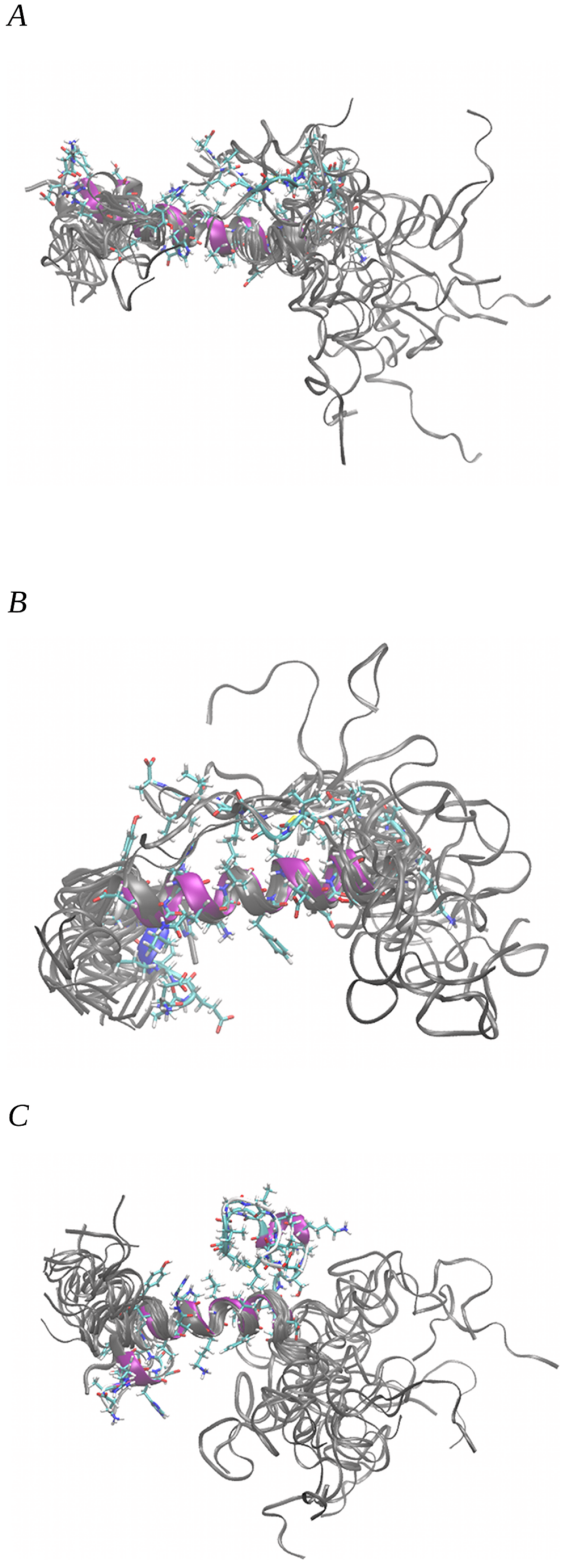
Representative structures of 20 clusters from MD simulations of amyloid with human amino acid sequence. Structure representing the most populated cluster is depicted as a licorice model. Figures A, B, and C are for simulations with 0.00, 0.15 and 0.30 M NaCl, respectively. For details on cluster populations, see Table 2.

**Figure 8:**
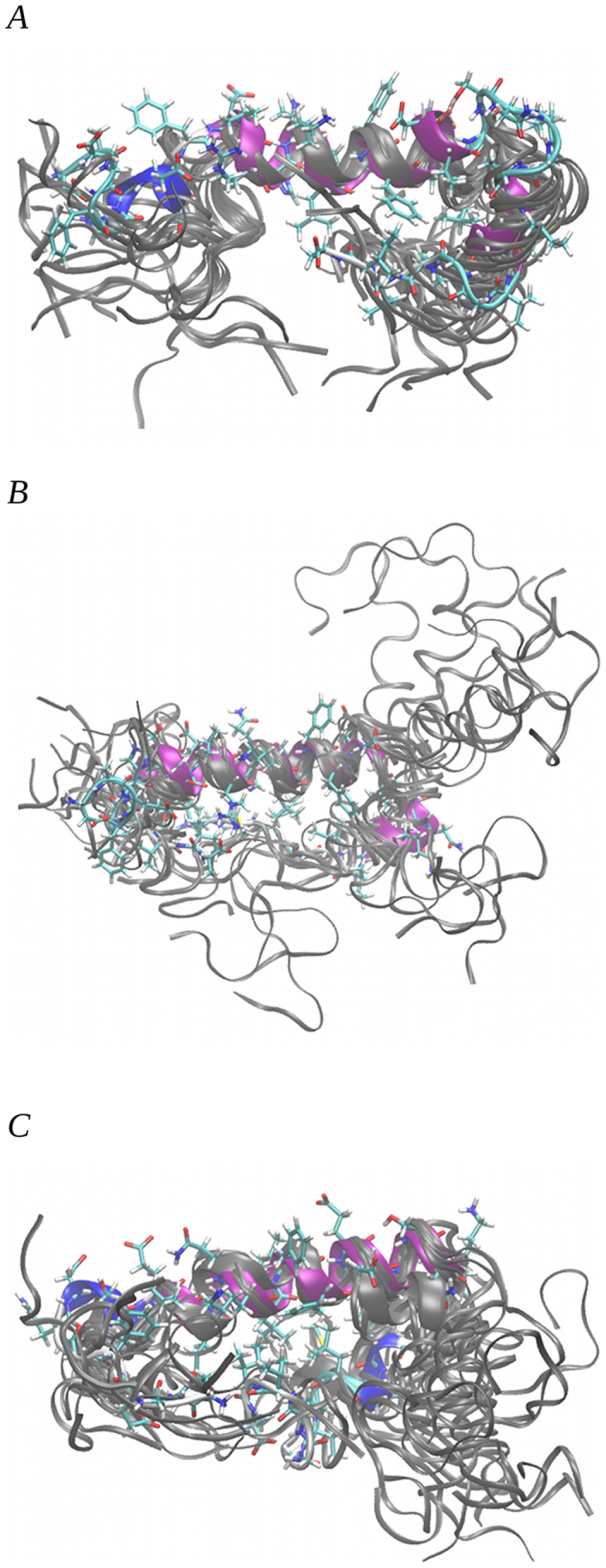
Representative structures of 20 clusters from MD simulations of amyloid with rat amino acids sequence. Structure representing the most populated cluster is depicted as a licorice model. Figures A, B, and C are for simulations with 0.00, 0.15 and 0.30 M NaCl, respectively. For details on cluster populations, see Table 3.

**Figure 9:**
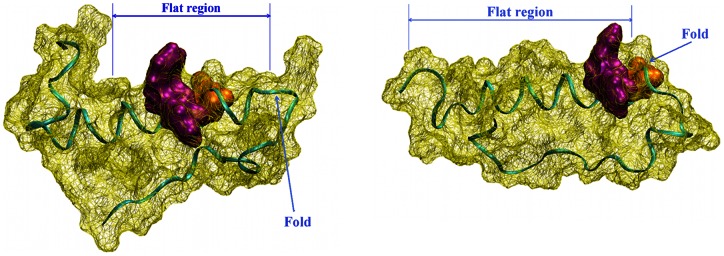
Solvent accessible surface area of representative conformations in most populated clusters of amyloid β peptide with phenylalanines 19 and 20 highlighted in violet and glutamic acid 22 in orange. Amyloid β peptide with human sequence – left figure and rat sequence – right figure. For more details, see the text.

Superpositions of representative conformers of all 20 clusters with alignment of residues from TYR/PHE10 to ALA21 (see Figure 7 for human and Figure 8 rat amyloid, respectively) showed that most of the populated structures are folded into globular conformations. The amyloid with the human amino acid sequence exhibited larger conformational changes than the rat amyloid. All human amyloid structures showed a fold between residues ASN27 and ILE32, which was predicted to be necessary for oligomer formation [41]. In the rat amyloid, this fold was observed near the hydrophobic core between residues ASP23 and GLY29. This fold was evoked by the formation of a strong hydrogen bond between the ALA42 backbone and ARG13 side chain, which was not possible in the human amyloid. This shift in the fold changes the shape of the amyloid surface and moves the position of PHE 19 and 20 together with GLU22 from a relatively narrow part of the surface in the human amyloid to the turn in the rat amyloid. The mutual orientation of phenylalanines and glutamic acid side chains play an important role in fibril formation [40]. The situation is demonstrated in Figure 9.

## Conclusions

In this study we aimed to elucidate the effect of ionic strength on the conformational behavior of amyloid beta peptides with human and rat amino acid sequences using molecular dynamics simulations.

We found the fold between residues ASN27 and ILE32 in all simulations of human amyloid peptide, which is in agreement with experimental results. In the rat amyloid peptide, this fold is located between residues VAL24 and GLY29. The shift in the described fold in the rat amyloid leads to the phenylalanines at positions 19 and 20 being moved from a relatively narrow region of the surface near the fold. These two phenylalanines play an important role in fibril formation together with GLU22 [40]. In the rat amyloid, the PHE 19 and 20 and GLU22 side chains are shifted to the turn region. This could be the reason for the differences in the fibril formation of both amyoids and may explain why the rat amyloid peptide does not make fibrils and plaques like the human amyloid.

We also found a destabilization of intramolecular hydrogen bonds caused by increasing the ionic strength of the solvent. This observation is in agreement with experimental data of amyloid oligomerization [21]. The destabilization of hydrogen bonds leads to an increase in flexibility and the formation of random coil or turn secondary structural elements, especially at the C-end of the peptide. The last third of the amyloid plays a crucial role in oligomer formation [42].

Our results gave a very stable α-helical secondary structure in all simulations. This is consistent with the beta-barrel models of amyloid oligomers published by Shafrir *et al.* [42], where the middle part of amyloid peptide exhibits a helical secondary structure. This stable part is also suggested to be a self-recognition part for oligomerization [5], [6].

Our analyses show that higher ionic strength destabilizes the intramolecular hydrogen bonds and leads to conformational changes in amino acid side chains that decrease the hydrophobic parts of the solvent-accessible surface area. This demonstrates the role of electrostatic and hydrophobic interactions on the conformational behavior of the peptides. These results are in agreement with studies performed on oligomers and fibrils [43]. The general behavior of both human and rat amyloids in their hydrophobic vs. hydrophilic SASA parts is similar in all simulations with different NaCl concentrations. Replacing the charged hydrophilic ARG5 with hydrophobic GLY5, hydrophilic TYR10 with hydrophobic PHE10 and charged hydrophilic HIS13 with charged hydrophilic ARG13 led to a decrease in the hydrophilic part of the SASA in the rat amyloid. The hydrophobic part of SASA values were similar in all simulations. Hydrophobicity is predicted to be one of the driving processes in fibril formation [41], but in this case the leading factor for destabilization of fibrils should be the changes in the shapes of monomeric units due to stabilization by the H-bond between the ALA42 C-end and ARG13 side chain, together with the shift of PHE19 and 20 and GLU22 to the turn of the amyloid structure in the rat amyloid.

## Supporting Information

Figure S1 **Calculated radius of gyration for Aβ with human amino acid sequence (left) and Aβ with rat amino acid sequence (right).** Graphs A, B, and C represent simulations with NaCl concentrations of 0.00, 0.15, and 0.30 M, respectively. The first MD run is represented by black color and the second MD run by grey color.
(TIFF)

Figure S2 **Time-dependent Ramachandran diagrams for all residues of Aβ with human amino acid sequence (residues 2 to 21) for simulations with NaCl concentrations of 0.00** **M.** The first quarter of the trajectory is depicted in yellow, second quarter in orange, third quarter in green and the last quarter in red.
(TIFF)

Figure S3 **Time-dependent Ramachandran diagrams for all residues of Aβ with human amino acid sequence (residues 22 to 41) for simulations with NaCl concentrations of 0.00** **M.** The first quarter of the trajectory is depicted in yellow, second quarter in orange, third quarter in green and the last quarter in red.
(TIFF)

Figure S4 **Time-dependent Ramachandran diagrams for all residues of Aβ with human amino acid sequence (residues 2 to 21) for simulations with NaCl concentrations of 0.15** **M.** The first quarter of the trajectory is depicted in yellow, second quarter in orange, third quarter in green and the last quarter in red.
(TIFF)

Figure S5 **Time-dependent Ramachandran diagrams for all residues of Aβ with human amino acid sequence (residues 22 to 41) for simulations with NaCl concentrations of 0.15** **M.** The first quarter of the trajectory is depicted in yellow, second quarter in orange, third quarter in green and the last quarter in red.
(TIFF)

Figure S6 **Time-dependent Ramachandran diagrams for all residues of Aβ with human amino acid sequence (residues 2 to 21) for simulations with NaCl concentrations of 0.30** **M.** The first quarter of the trajectory is depicted in yellow, second quarter in orange, third quarter in green and the last quarter in red.
(TIFF)

Figure S7 **Time-dependent Ramachandran diagrams for all residues of Aβ with human amino acid sequence (residues 22 to 41) for simulations with NaCl concentrations of 0.30** **M.** The first quarter of the trajectory is depicted in yellow, second quarter in orange, third quarter in green and the last quarter in red.
(TIFF)

Figure S8 **Time-dependent Ramachandran diagrams for all residues of Aβ with rat amino acid sequence (residues 2 to 21) for simulations with NaCl concentrations of 0.00** **M.** The first quarter of trajectory is depicted in yellow, second quarter in orange, third quarter in green and the last quarter in red.
(TIFF)

Figure S9 **Time-dependent Ramachandran diagrams for all residues of Aβ with rat amino acid sequence (residues 22 to 41) for simulations with NaCl concentrations of 0.00** **M.** The first quarter of trajectory is depicted in yellow, second quarter in orange, third quarter in green and the last quarter in red.
(TIFF)

Figure S10 **Time-dependent Ramachandran diagrams for all residues of Aβ with rat amino acid sequence (residues 2 to 21) for simulations with NaCl concentrations of 0.15** **M.** The first quarter of trajectory is depicted in yellow, second quarter in orange, third quarter in green and the last quarter in red.
(TIFF)

Figure S11 **Time-dependent Ramachandran diagrams for all residues of Aβ with rat amino acid sequence (residues 22 to 41) for simulations with NaCl concentrations of 0.15** **M.** The first quarter of trajectory is depicted in yellow, second quarter in orange, third quarter in green and the last quarter in red.
(TIFF)

Figure S12 **Time-dependent Ramachandran diagrams for all residues of Aβ with rat amino acid sequence (residues 2 to 21) for simulations with NaCl concentrations of 0.30** **M.** The first quarter of trajectory is depicted in yellow, second quarter in orange, third quarter in green and the last quarter in red.
(TIFF)

Figure S13 **Time-dependent Ramachandran diagrams for all residues of Aβ with rat amino acid sequence (residues 22 to 41) for simulations with NaCl concentrations of 0.30** **M.** The first quarter of trajectory is depicted in yellow, second quarter in orange, third quarter in green and the last quarter in red.
(TIFF)

Table S1 **Most significant intramolecular hydrogen bonds with occupancy greater than 50% of the trajectory and their geometric characteristics (donor-acceptor distance, acceptor-donor-hydrogen angles) found for amyloid with human amino acid sequence calculated from molecular dynamics simulation for c(NaCl)  = 0.00** **M.**
(DOC)

Table S2 **Most significant intramolecular hydrogen bonds with occupancy greater than 50% of the trajectory and their geometric characteristics (donor-acceptor distance, acceptor-donor-hydrogen angles) found for amyloid with human amino acid sequence calculated from molecular dynamics simulation for c(NaCl)  = 0.15** **M.**
(DOC)

Table S3 **Most significant intramolecular hydrogen bonds with occupancy greater than 50% of the trajectory and their geometric characteristics (donor-acceptor distance, acceptor-donor-hydrogen angles) found for amyloid with human amino acid sequence calculated from molecular dynamics simulation for c(NaCl)  = 0.30** **M.**
(DOC)

Table S4 **Most significant intramolecular hydrogen bonds with occupancy greater than 50% of the trajectory and their geometric characteristics (donor-acceptor distance, acceptor-donor-hydrogen angles) found for amyloid with rat amino acid sequence calculated from molecular dynamics simulation for c(NaCl)  = 0.00** **M.**
(DOC)

Table S5 **Most significant intramolecular hydrogen bonds with occupancy greater than 50% of the trajectory and their geometric characteristics (donor-acceptor distance, acceptor-donor-hydrogen angles) found for amyloid with rat amino acid sequence calculated from molecular dynamics simulation for c(NaCl)  = 0.15** **M.**
(DOC)

Table S6 **Most significant intramolecular hydrogen bonds with occupancy greater than 50% of the trajectory and their geometric characteristics (donor-acceptor distance, acceptor-donor-hydrogen angles) found for amyloid with rat amino acid sequence calculated from molecular dynamics simulation for c(NaCl)  = 0.30** **M.**
(DOC)

Cluster S1 **Coordinate files.** Cartesian coordinates of representative structures in pdb format are included in the file Cluster S1. Coordinates are stored in subdirectories for human amyloid with NaCl concentration of 0.00 M (cluster_human_0 M), 0.15 M (cluster_human_015) and 0.30 M (cluster_human_030 M). Rat amyloid data are stored in subdirectories: cluster_rat_0 M, cluster_rat_015 M and cluster_rat_030 M.
(ZIP)
