# Supplementary figures and images for: How Ionic Strength Affects the Conformational Behavior of Human and Rat Beta Amyloids – A Computational Study
Source: PLoS One. 2013 May 23;8(5):e62914. doi: 10.1371/journal.pone.0062914 (PMC3662769; doi:10.1371/journal.pone.0062914)

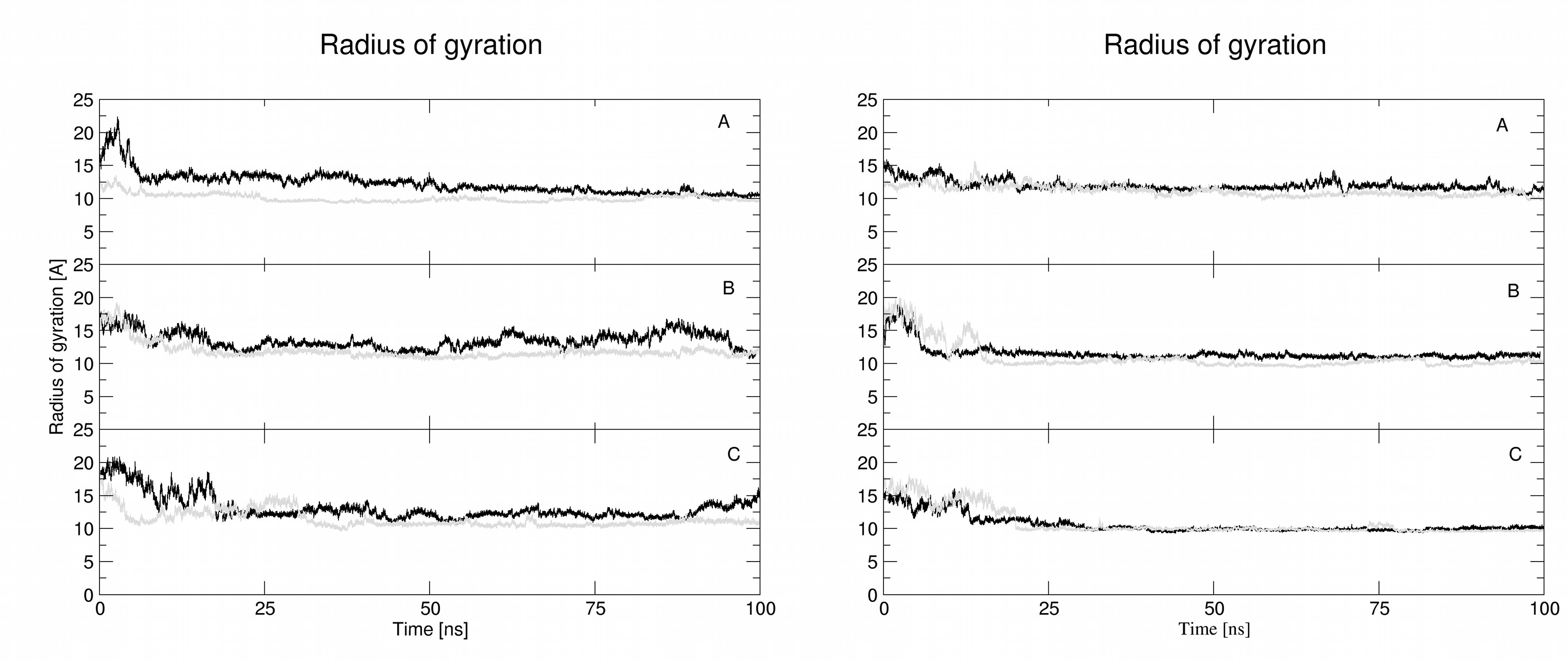

Supplement: Figure S1 — Calculated radius of gyration for Aβ with human amino acid sequence (left) and Aβ with rat amino acid sequence (right). Graphs A, B, and C represent simulations with NaCl concentrations of 0.00, 0.15, and 0.30 M, respectively. The first MD run is represented by black color and the second MD run by grey color. (TIFF) [file pone.0062914.s001.tiff]

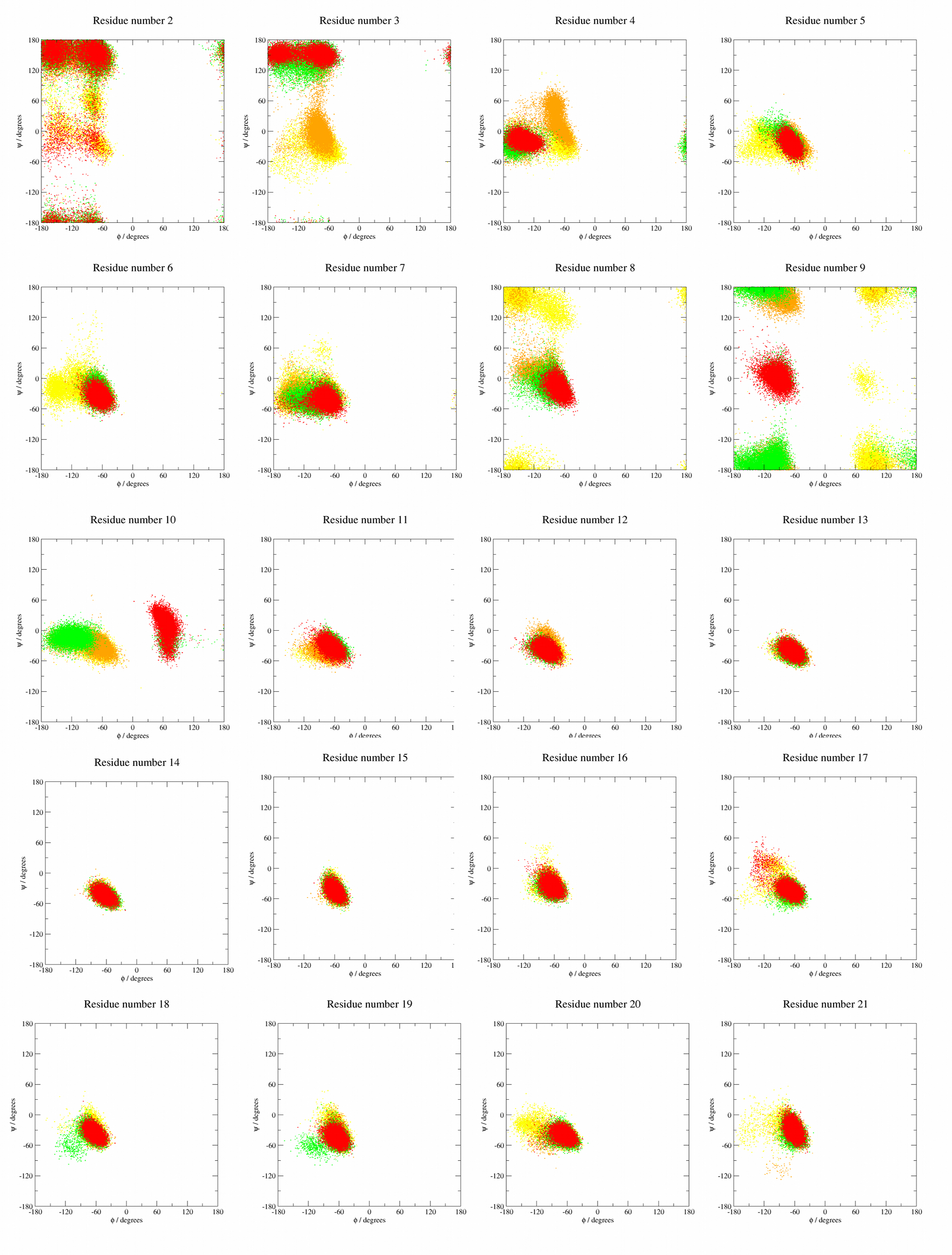

Supplement: Figure S2 — Time-dependent Ramachandran diagrams for all residues of Aβ with human amino acid sequence (residues 2 to 21) for simulations with NaCl concentrations of 0.00 M. The first quarter of the trajectory is depicted in yellow, second quarter in orange, third quarter in green and the last quarter in red. (TIFF) [file pone.0062914.s002.tiff]

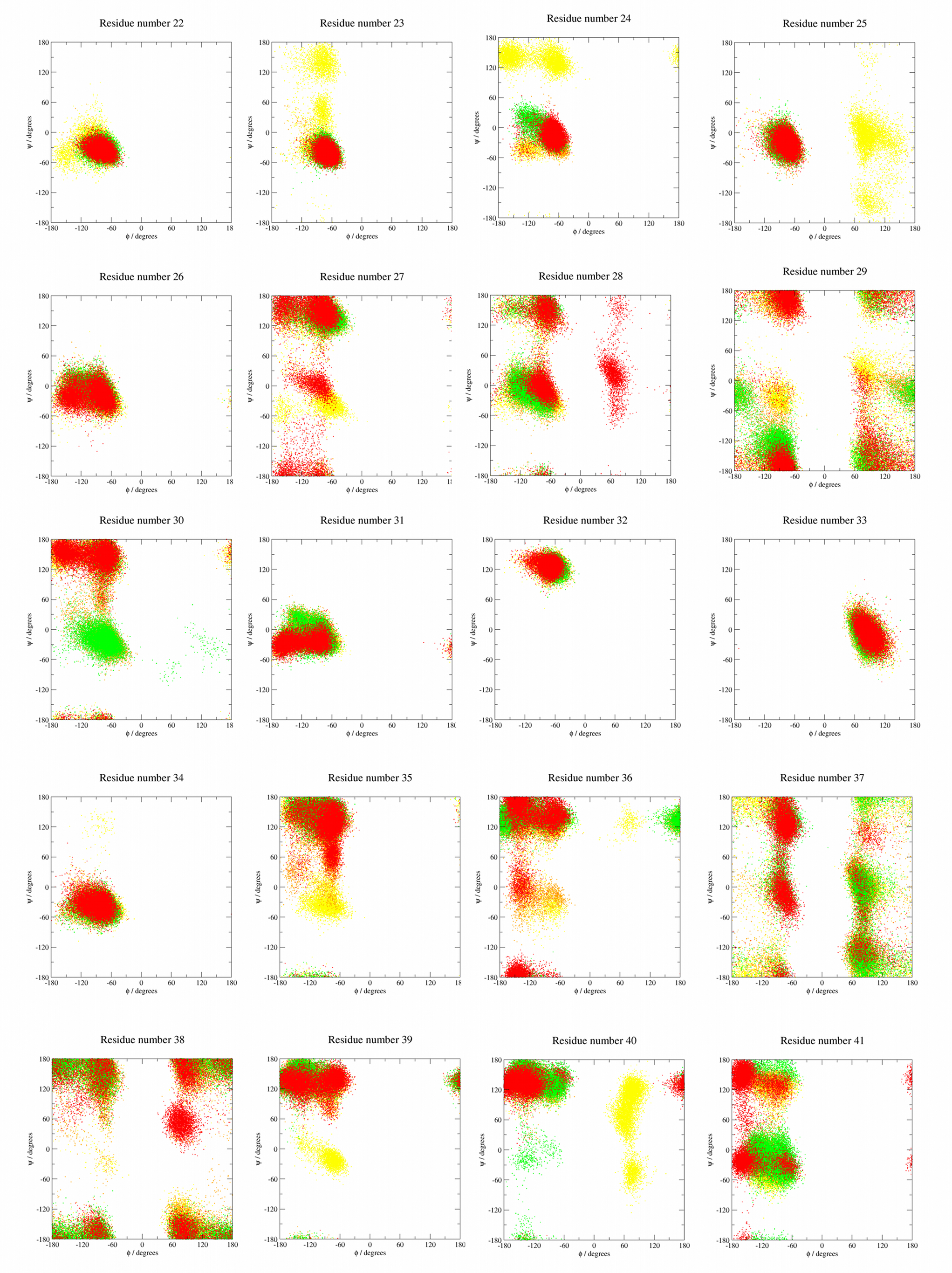

Supplement: Figure S3 — Time-dependent Ramachandran diagrams for all residues of Aβ with human amino acid sequence (residues 22 to 41) for simulations with NaCl concentrations of 0.00 M. The first quarter of the trajectory is depicted in yellow, second quarter in orange, third quarter in green and the last quarter in red. (TIFF) [file pone.0062914.s003.tiff]

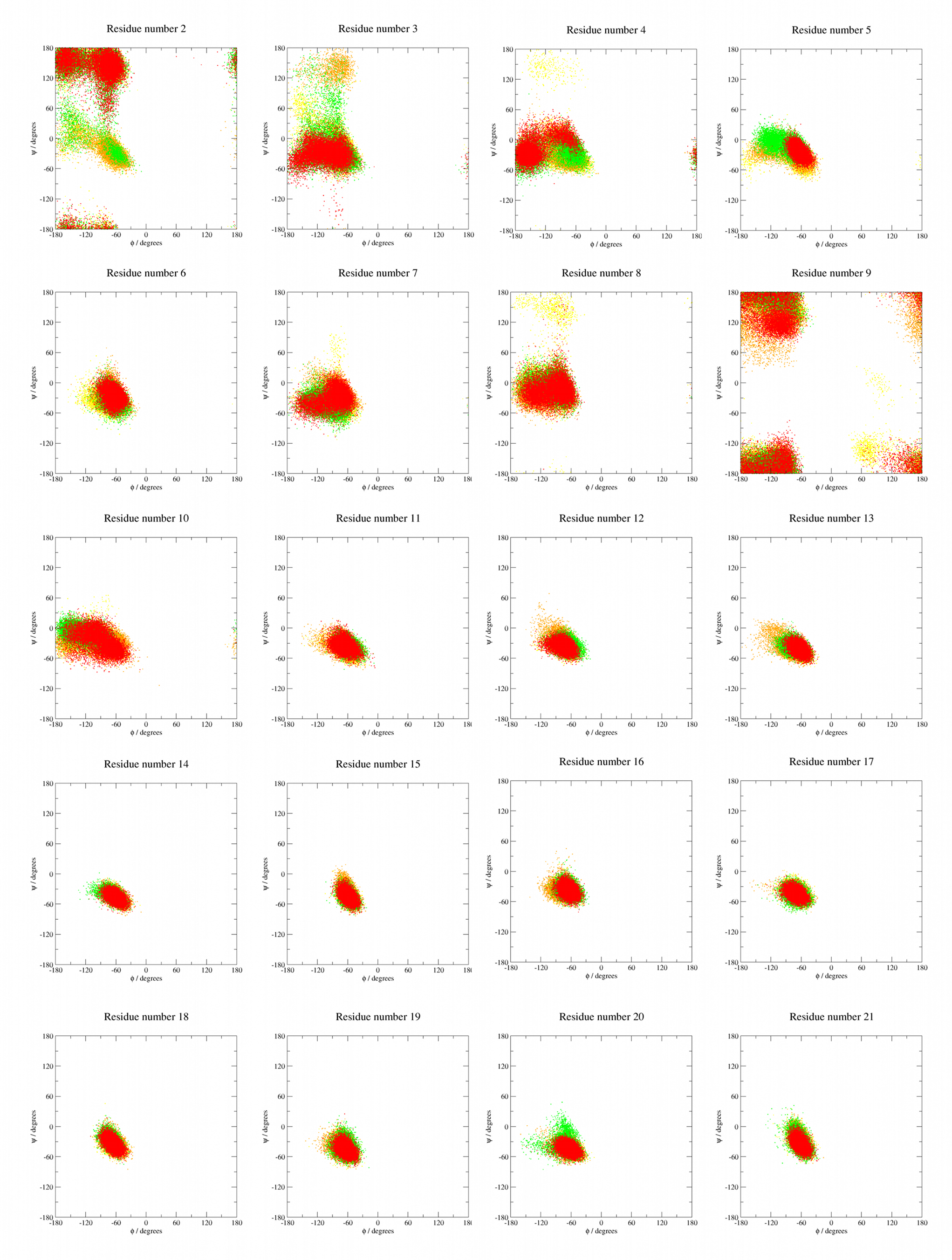

Supplement: Figure S4 — Time-dependent Ramachandran diagrams for all residues of Aβ with human amino acid sequence (residues 2 to 21) for simulations with NaCl concentrations of 0.15 M. The first quarter of the trajectory is depicted in yellow, second quarter in orange, third quarter in green and the last quarter in red. (TIFF) [file pone.0062914.s004.tiff]

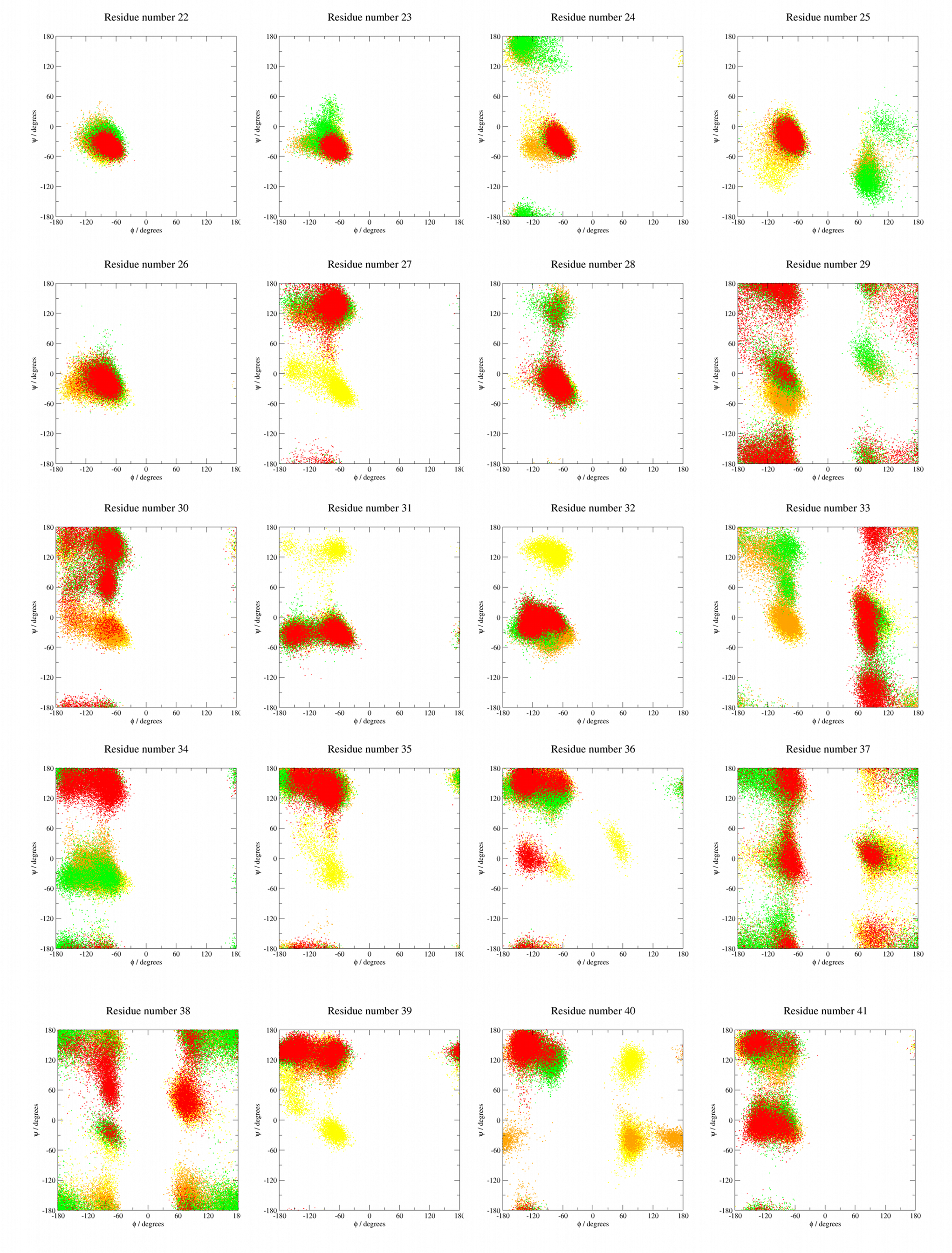

Supplement: Figure S5 — Time-dependent Ramachandran diagrams for all residues of Aβ with human amino acid sequence (residues 22 to 41) for simulations with NaCl concentrations of 0.15 M. The first quarter of the trajectory is depicted in yellow, second quarter in orange, third quarter in green and the last quarter in red. (TIFF) [file pone.0062914.s005.tiff]

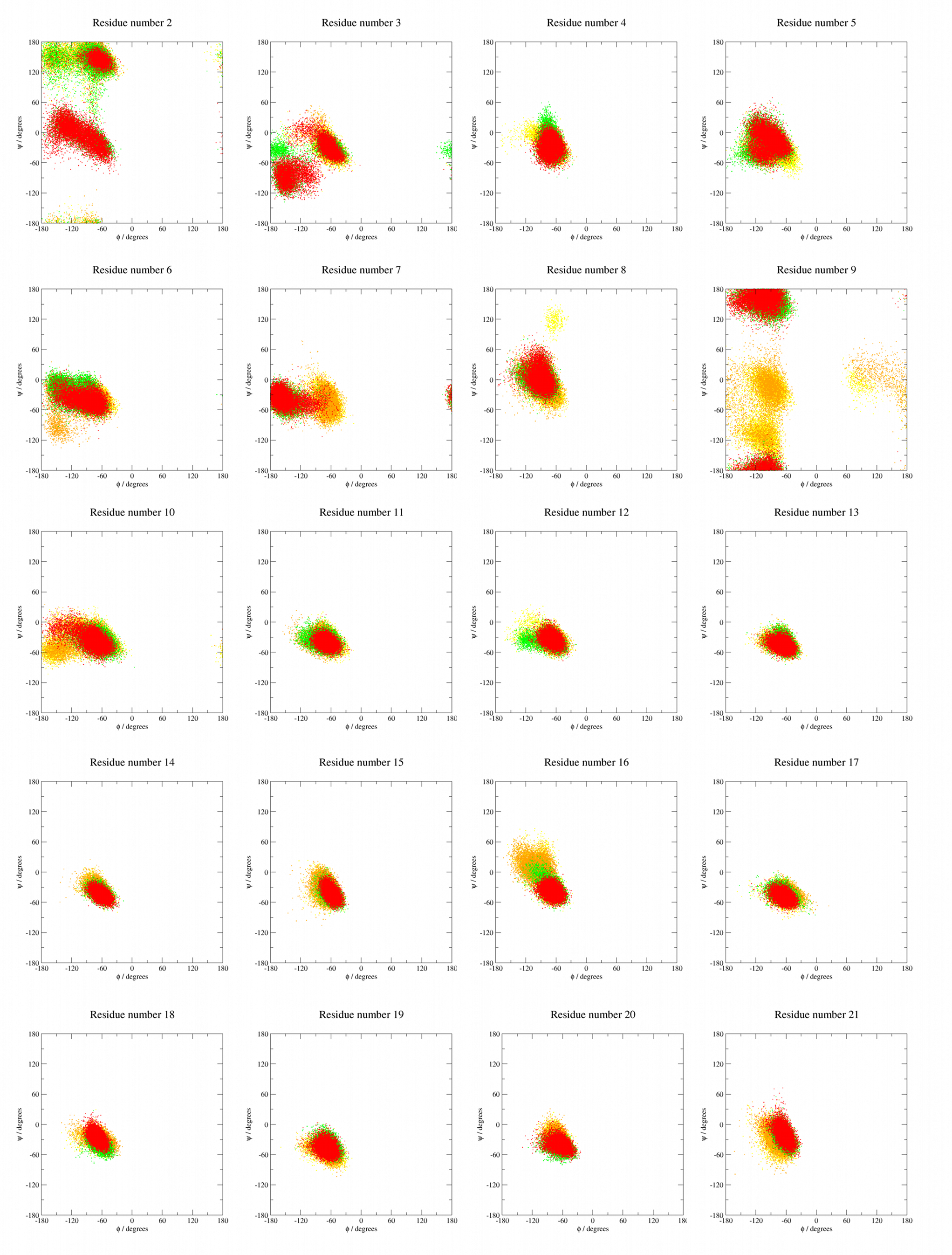

Supplement: Figure S6 — Time-dependent Ramachandran diagrams for all residues of Aβ with human amino acid sequence (residues 2 to 21) for simulations with NaCl concentrations of 0.30 M. The first quarter of the trajectory is depicted in yellow, second quarter in orange, third quarter in green and the last quarter in red. (TIFF) [file pone.0062914.s006.tiff]

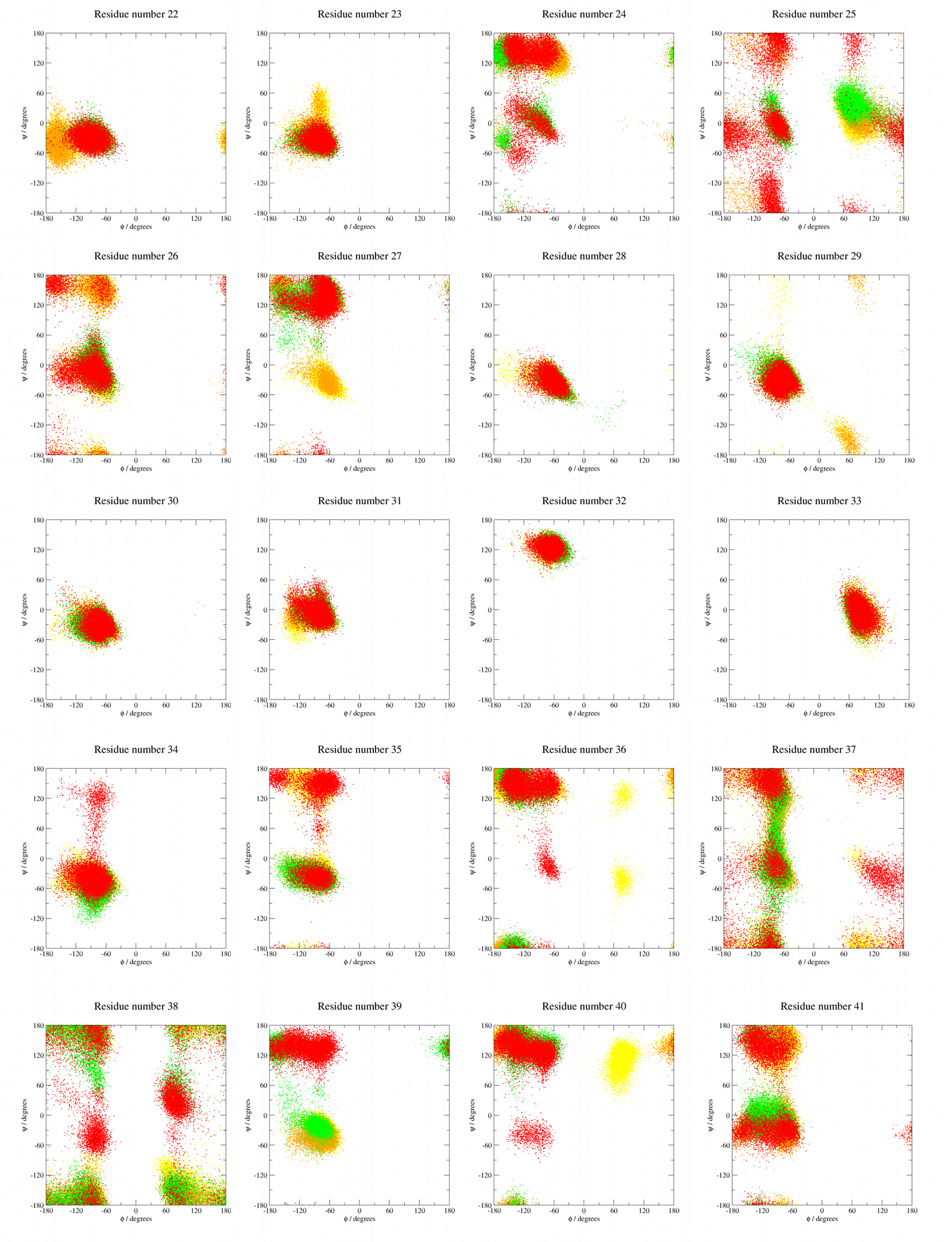

Supplement: Figure S7 — Time-dependent Ramachandran diagrams for all residues of Aβ with human amino acid sequence (residues 22 to 41) for simulations with NaCl concentrations of 0.30 M. The first quarter of the trajectory is depicted in yellow, second quarter in orange, third quarter in green and the last quarter in red. (TIFF) [file pone.0062914.s007.tiff]

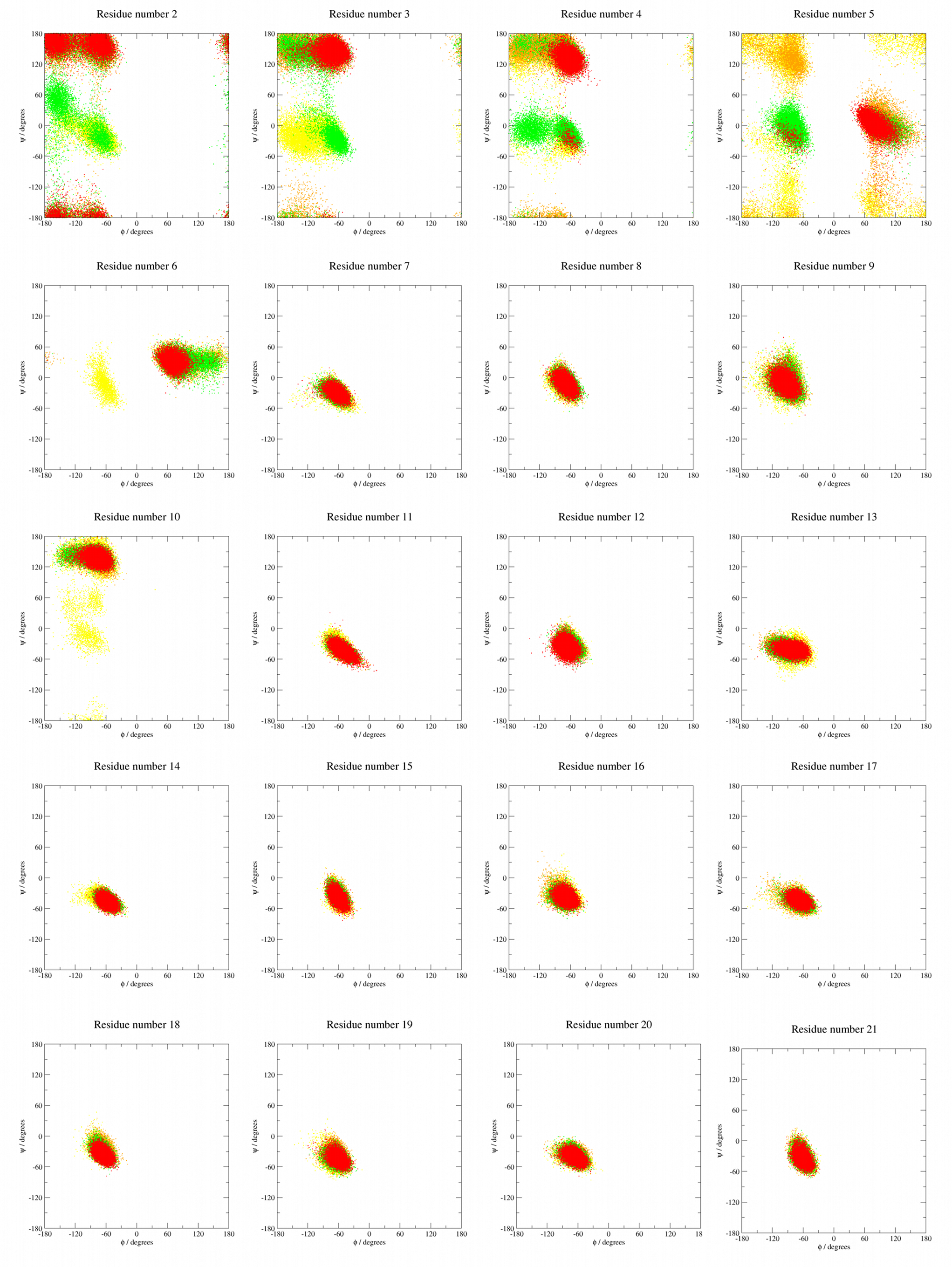

Supplement: Figure S8 — Time-dependent Ramachandran diagrams for all residues of Aβ with rat amino acid sequence (residues 2 to 21) for simulations with NaCl concentrations of 0.00 M. The first quarter of trajectory is depicted in yellow, second quarter in orange, third quarter in green and the last quarter in red. (TIFF) [file pone.0062914.s008.tiff]

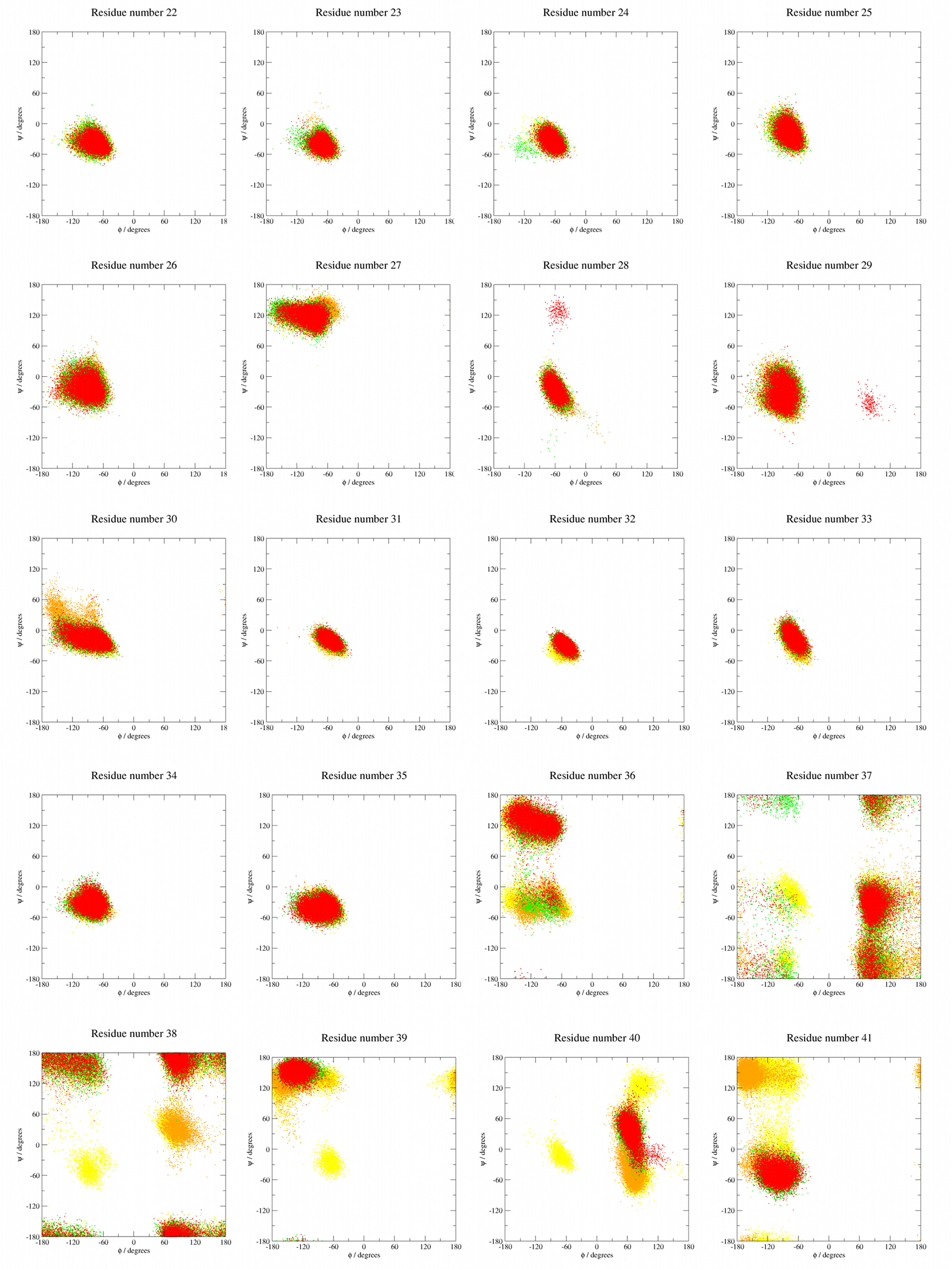

Supplement: Figure S9 — Time-dependent Ramachandran diagrams for all residues of Aβ with rat amino acid sequence (residues 22 to 41) for simulations with NaCl concentrations of 0.00 M. The first quarter of trajectory is depicted in yellow, second quarter in orange, third quarter in green and the last quarter in red. (TIFF) [file pone.0062914.s009.tiff]

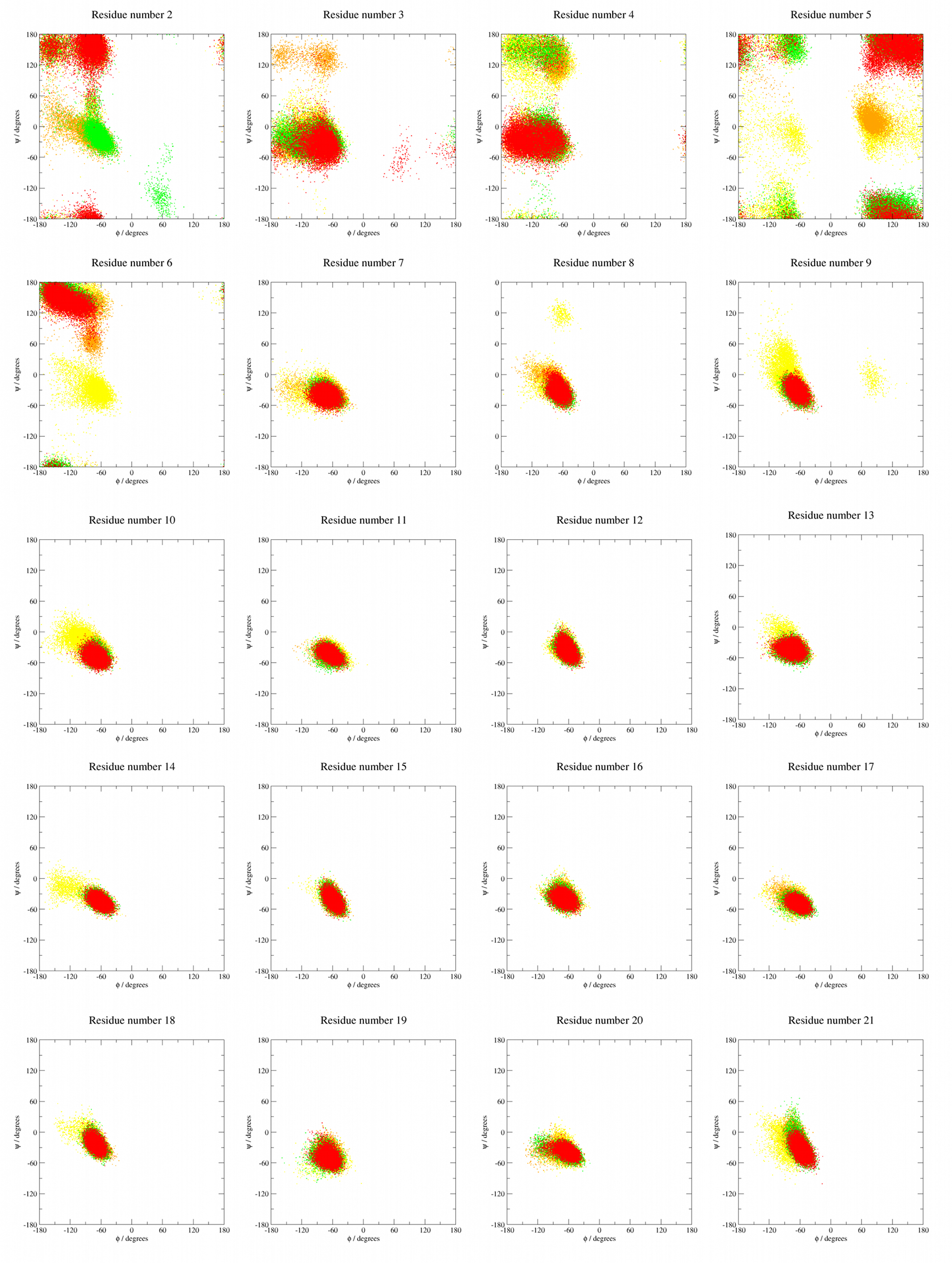

Supplement: Figure S10 — Time-dependent Ramachandran diagrams for all residues of Aβ with rat amino acid sequence (residues 2 to 21) for simulations with NaCl concentrations of 0.15 M. The first quarter of trajectory is depicted in yellow, second quarter in orange, third quarter in green and the last quarter in red. (TIFF) [file pone.0062914.s010.tiff]

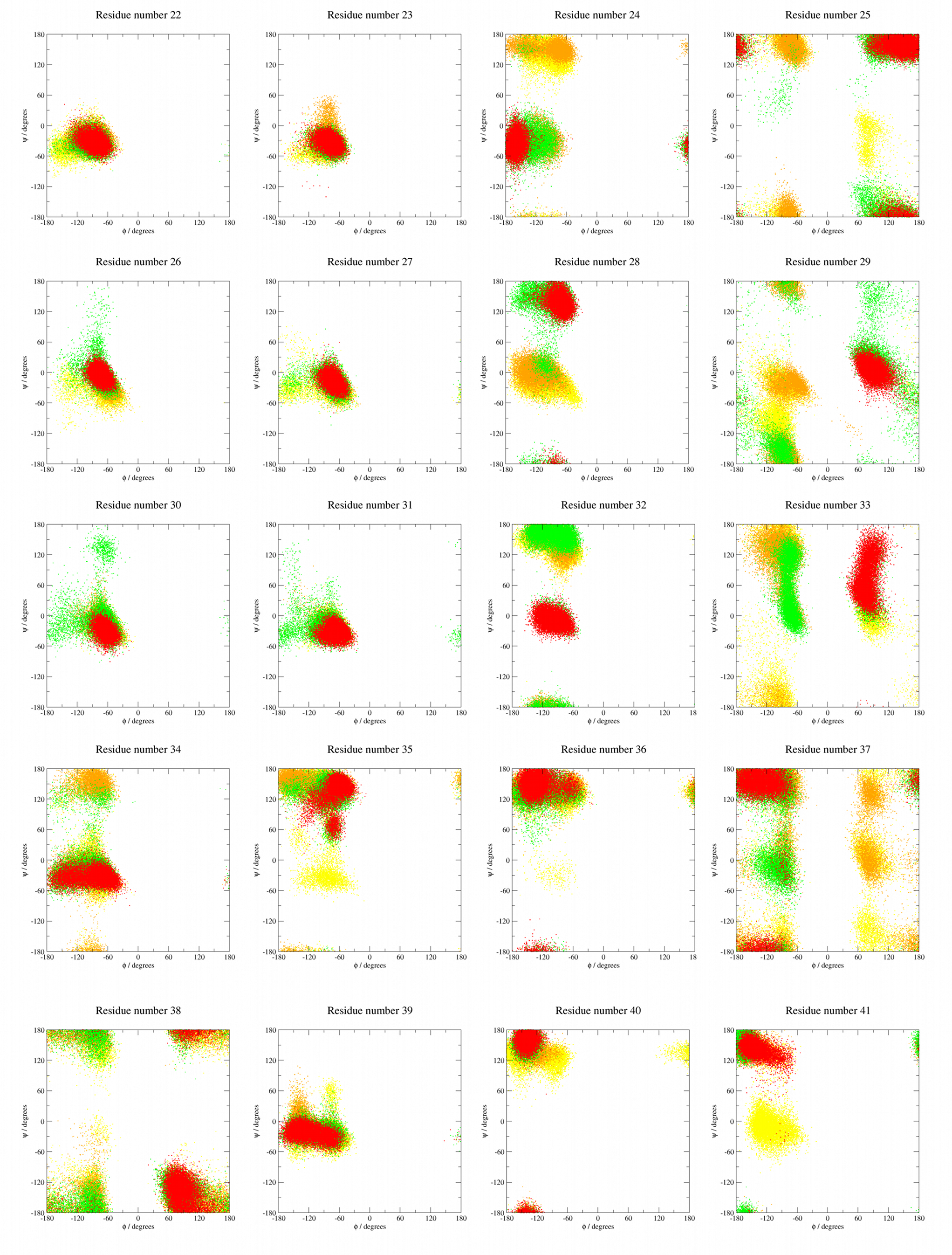

Supplement: Figure S11 — Time-dependent Ramachandran diagrams for all residues of Aβ with rat amino acid sequence (residues 22 to 41) for simulations with NaCl concentrations of 0.15 M. The first quarter of trajectory is depicted in yellow, second quarter in orange, third quarter in green and the last quarter in red. (TIFF) [file pone.0062914.s011.tiff]

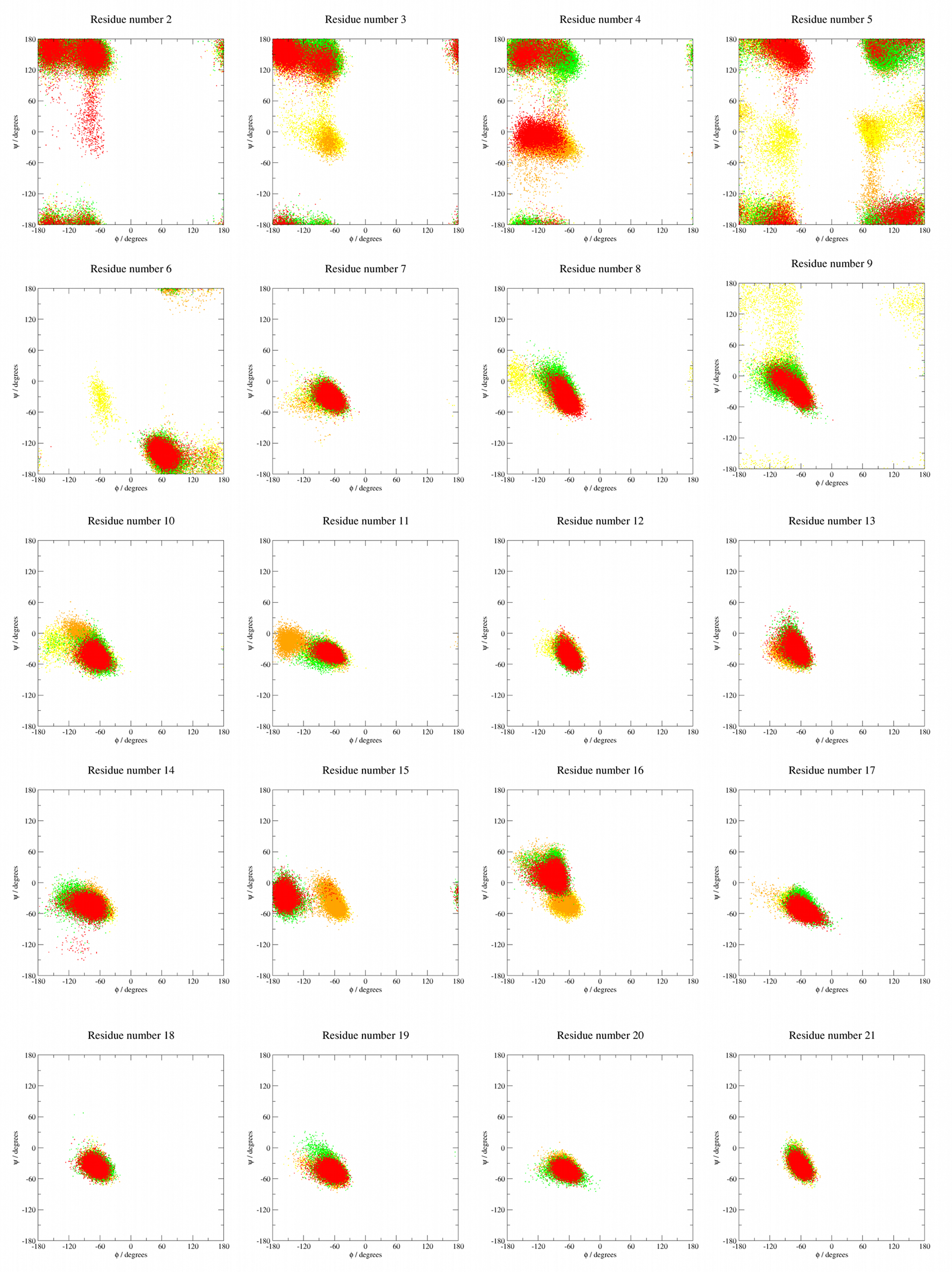

Supplement: Figure S12 — Time-dependent Ramachandran diagrams for all residues of Aβ with rat amino acid sequence (residues 2 to 21) for simulations with NaCl concentrations of 0.30 M. The first quarter of trajectory is depicted in yellow, second quarter in orange, third quarter in green and the last quarter in red. (TIFF) [file pone.0062914.s012.tiff]

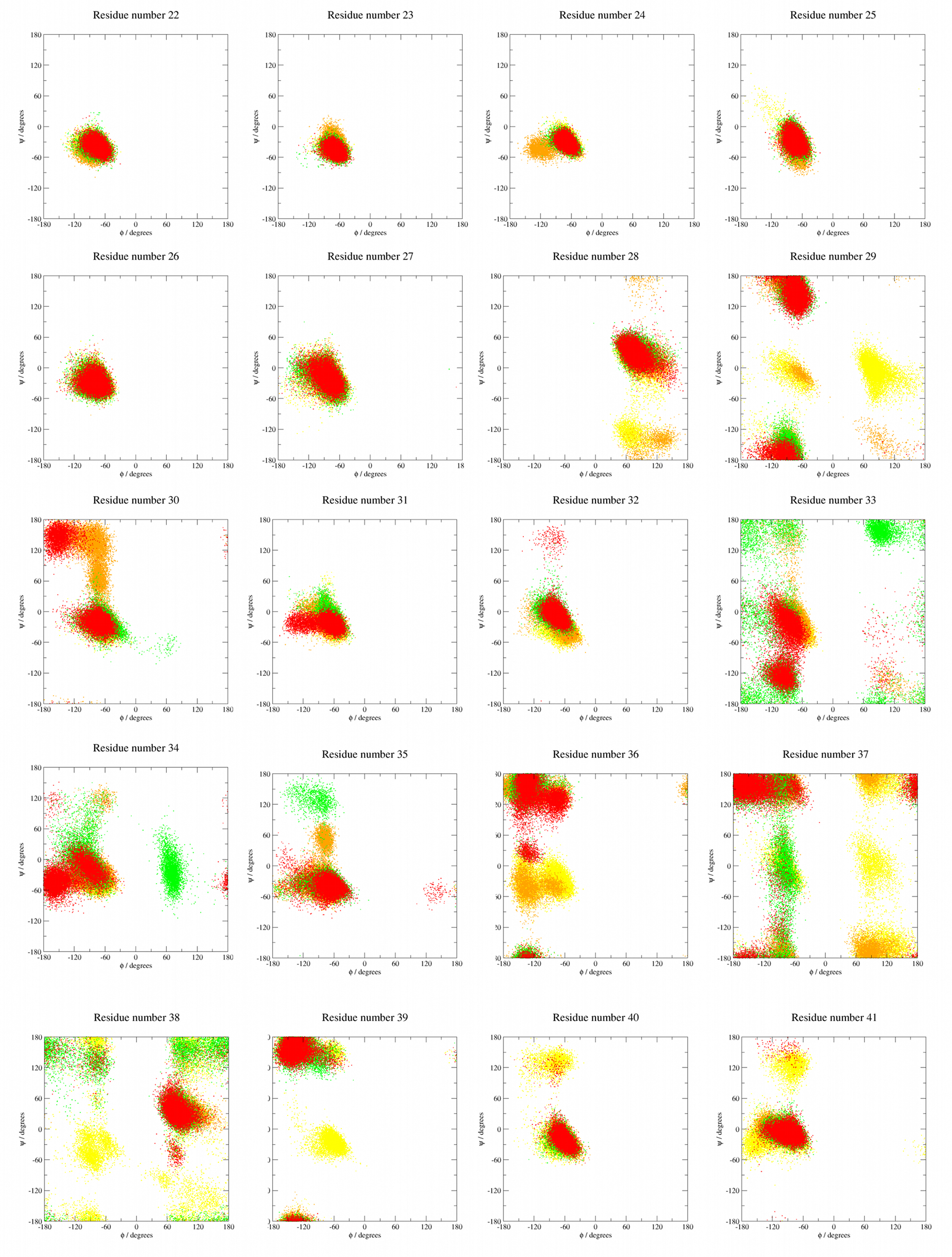

Supplement: Figure S13 — Time-dependent Ramachandran diagrams for all residues of Aβ with rat amino acid sequence (residues 22 to 41) for simulations with NaCl concentrations of 0.30 M. The first quarter of trajectory is depicted in yellow, second quarter in orange, third quarter in green and the last quarter in red. (TIFF) [file pone.0062914.s013.tiff]
