# Supplementary material for: How Ionic Strength Affects the Conformational Behavior of Human and Rat Beta Amyloids – A Computational Study
Source: PLoS One. 2013 May 23;8(5):e62914. doi: 10.1371/journal.pone.0062914 (PMC3662769; doi:10.1371/journal.pone.0062914)
Supplement: Table S1 — Most significant intramolecular hydrogen bonds with occupancy greater than 50% of the trajectory and their geometric characteristics (donor-acceptor distance, acceptor-donor-hydrogen angles) found for amyloid with human amino acid sequence calculated from molecular dynamics simulation for c(NaCl) = 0.00 M. (DOC) [file pone.0062914.s014.doc]

**Table S1:**

Most significant intramolecular hydrogen bonds with occupancy large than 50 % of the trajectory and their geometric characteristics found for amyloid with human amino acids sequence calculated from molecular dynamics simulation for c(NaCl) = 0.00 mol.dm^-3^.

| Acceptor | Donor - H | Occupancy [%] | Distance D-A [Å] | Angle A-D-H [degrees] |
| --- | --- | --- | --- | --- |
| O (LEU17) | N-H (ALA21) | 96.92 | 2.96±0.16 | 18.96±10.91 |
| O (HIS14) | N-H (VAL18) | 96.75 | 2.88±0.13 | 17.54±10.57 |
| O (HIS13) | N-H (LEU17) | 93.06 | 2.99±0.16 | 27.71±13.11 |
| O (GLU11) | N-H (GLN15) | 92.71 | 3.03±0.18 | 22.20±11.25 |
| O (TYR10) | N-H (HIS14) | 90.52 | 3.01±0.18 | 21.04±11.49 |
| O (PHE20) | N-H (VAL24) | 88.43 | 2.94±0.15 | 16.98±10.10 |
| O (PHE19) | N-H (ASP23) | 79.84 | 3.04±0.18 | 23.22±11.04 |
| O (VAl12) | N-H (LYS16) | 76.36 | 3.11±0.18 | 19.22±11.04 |
| O(PHE4) | N-H (SER8) | 71.75 | 3.02±0.19 | 20.70±11.95 |
| O (GLN15) | N-H (PHE19) | 69.15 | 3.06±0.18 | 23.64±12.09 |
| O (VAL18) | N-H (GLU22) | 67.97 | 3.09±0.19 | 29.04±14.11 |
| O (LYS16) | N-H (PHE20) | 64.53 | 3.10±0.19 | 24.30±12.70 |
| O (PHE4) | N-H (ASP7) | 55.45 | 3.10±0.18 | 31.10±13.75 |
| O (ILE31) | N-H (LEU34) | 55.07 | 3.13±0.18 | 30.80±11.90 |
| O (GLU22) | OG-HG (SER26) | 52.64 | 2.74 ± 0.18 | 18.00 ± 10.62 |
