# Supplementary material for: How Ionic Strength Affects the Conformational Behavior of Human and Rat Beta Amyloids – A Computational Study
Source: PLoS One. 2013 May 23;8(5):e62914. doi: 10.1371/journal.pone.0062914 (PMC3662769; doi:10.1371/journal.pone.0062914)
Supplement: Table S2 — Most significant intramolecular hydrogen bonds with occupancy greater than 50% of the trajectory and their geometric characteristics (donor-acceptor distance, acceptor-donor-hydrogen angles) found for amyloid with human amino acid sequence calculated from molecular dynamics simulation for c(NaCl) = 0.15 M. (DOC) [file pone.0062914.s015.doc]

**Table S2:**

Most significant intramolecular hydrogen bonds with occupancy large than 50 % of the trajectory and their geometric characteristics found for amyloid with human amino acids sequence calculated from molecular dynamics simulation for c(NaCl) = 0.15 mol.dm^-3^.

| Acceptor | Donor - H | Occupancy [%] | Distance D-A [Å] | Angle A-D-H [degrees] |
| --- | --- | --- | --- | --- |
| O (HIS14) | N-H (VAL18) | 99.52 | 2.91±0.14 | 16.79±9.95 |
| O (LEU17) | N-H (ALA21) | 96.41 | 2.96±0.16 | 19.21±10.70 |
| O (HIS13) | N-H (LEU17) | 95.71 | 3.01±0.17 | 26.25±12.93 |
| O (PHE20) | N-H (VAL24) | 93.52 | 2.89±0.13 | 18.21±10.47 |
| O (GLU11) | N-H (GLN15) | 89.17 | 3.01±0.18 | 20.60±10.80 |
| O (TYR10) | N-H (HIS14) | 87.00 | 3.00±0.18 | 21.02±11.34 |
| O (PHE19) | N-H (ASP23) | 81.40 | 3.04±0.18 | 25.60±13.60 |
| O (LYS16) | N-H (PHE20) | 80.53 | 3.10±0.19 | 23.65±12.49 |
| O (GLN15) | N-H (PHE19) | 79.42 | 3.08±0.19 | 25.56±12.50 |
| O (VAL12) | N-H (LYS16) | 77.14 | 3.11±0.18 | 20.98±11.74 |
| O (VAL18) | N-H (GLU22) | 68.35 | 3.11±0.19 | 31.00±14.10 |
| OD (ASN27) | N-H (GLY29) | 58.54 | 3.06±0.18 | 32.01±13.72 |
| O (GLU22) | OG-HG (SER26) | 56.21 | 2.72±0.14 | 17.00±9.70 |
| O (PHE4) | N-H (ASP7) | 54.87 | 3.11±0.18 | 30.90±14.13 |
| O (PHE4) | N-H (SER8) | 53.84 | 2.99±0.18 | 23.80±13.25 |
| O (ARG5) | N-H (GLY9) | 53.07 | 3.03±0.20 | 36.94±14.95 |
| O (GLU11) | NE-HE (GLN15) | 52.28 | 3.00±0.18 | 23.56±10.95 |
| O (ALA21) | N-H (GLY25) | 51.29 | 3.06±0.19 | 37.83±12.70 |
