# Supplementary material for: How Ionic Strength Affects the Conformational Behavior of Human and Rat Beta Amyloids – A Computational Study
Source: PLoS One. 2013 May 23;8(5):e62914. doi: 10.1371/journal.pone.0062914 (PMC3662769; doi:10.1371/journal.pone.0062914)
Supplement: Table S3 — Most significant intramolecular hydrogen bonds with occupancy greater than 50% of the trajectory and their geometric characteristics (donor-acceptor distance, acceptor-donor-hydrogen angles) found for amyloid with human amino acid sequence calculated from molecular dynamics simulation for c(NaCl) = 0.30 M. (DOC) [file pone.0062914.s016.doc]

**Table S3:**

Most significant intramolecular hydrogen bonds with occupancy large than 50 % of the trajectory and their geometric characteristics found for amyloid with human amino acids sequence calculated from molecular dynamics simulation for c(NaCl) = 0.30 mol.dm^-3^.

| Acceptor | Donor - H | Occupancy [%] | Distance D-A [Å] | Angle A-D-H [degrees] |
| --- | --- | --- | --- | --- |
| O (LEU17) | N-H (ALA21) | 96.43 | 2.98±0.17 | 22.44±12.20 |
| O (GLU11) | N-H (GLN15) | 92.91 | 3.02±0.18 | 22.13±11.80 |
| O (TYR10) | N-H (HIS14) | 89.35 | 2.99±0.17 | 22.02±11.76 |
| O (GLU3) | N-H (ASP7) | 85.09 | 2.97±0.16 | 21.80±11.67 |
| O (HIS13) | N-H (LEU17) | 81.34 | 3.13±0.18 | 29.14±11.44 |
| O (ILE31) | N-H (LEU34) | 76.02 | 3.01±0.17 | 26.32±13.50 |
| O (ALA2) | N-H (ARG5) | 75.95 | 3.07±0.17 | 28.50±11.90 |
| O (VAL12) | N-H (LYS16) | 75.67 | 3.12±0.18 | 22.46±12.80 |
| O (HIS14) | N-H (VAL18) | 75.51 | 2.90±0.13 | 17.30±10.27 |
| O (LYS16) | N-H (PHE20) | 74.56 | 3.09±0.18 | 21.68±12.11 |
| O (PHE20) | N-H (VAL24) | 66.18 | 2.93±0.16 | 21.90±11.83 |
| O (GLU3) | N-H (SER8) | 62.19 | 2.93±0.15 | 23.54±12.47 |
| O (GLN15) | N-H (PHE19) | 60.09 | 3.09±0.19 | 25.75±12.68 |
| O (ASN27) | N-H (ILE31) | 58.14 | 3.14±0.18 | 21.36±11.72 |
| O (PHE19) | N-H (ASP23) | 57.45 | 3.04±0.19 | 31.02±15.20 |
| O (PHE19) | N-H (GLU22) | 55.98 | 3.16±0.19 | 36.83±12.16 |
| O (VAL18) | N-H (GLU22) | 55.03 | 3.12±0.18 | 35.26±14.42 |
| O (GLU3) | N-H (HIS6) | 50.02 | 3.15±0.17 | 34.91±14.05 |
