# Supplementary material for: How Ionic Strength Affects the Conformational Behavior of Human and Rat Beta Amyloids – A Computational Study
Source: PLoS One. 2013 May 23;8(5):e62914. doi: 10.1371/journal.pone.0062914 (PMC3662769; doi:10.1371/journal.pone.0062914)
Supplement: Table S4 — Most significant intramolecular hydrogen bonds with occupancy greater than 50% of the trajectory and their geometric characteristics (donor-acceptor distance, acceptor-donor-hydrogen angles) found for amyloid with rat amino acid sequence calculated from molecular dynamics simulation for c(NaCl) = 0.00 M. (DOC) [file pone.0062914.s017.doc]

**Table S4:**

Most significant intramolecular hydrogen bonds with occupancy larger than 50 % of the trajectory and their geometric characteristics found for amyloid with rat amino acids sequence calculated from molecular dynamics simulation for c(NaCl) = 0.00 mol.dm^-3^.

| Acceptor | Donor - H | Occupancy [%] | Distance D-A [Å] | Angle A-D-H [degrees] |
| --- | --- | --- | --- | --- |
| O (ILE32) | N-H (VAL36) | 99.79 | 2.81±0.11 | 18.64±10.12 |
| O (PHE20) | N-H (VAL24) | 99.23 | 2.88±0.13 | 17.11±9.65 |
| O (HIS14) | N-H (VAL18) | 99.11 | 2.93±0.14 | 14.65±8.41 |
| O (GLU11) | N-H (GLN15) | 96.13 | 3.00±0.18 | 15.36±7.96 |
| O (ILE31) | N-H (LEU34) | 94.93 | 3.01±0.16 | 30.22±12.13 |
| O (LEU17) | N-H (ALA21) | 93.57 | 2.99±0.17 | 21.40±11.02 |
| O (ARG13) | N-H (LEU17) | 92.90 | 3.05±0.17 | 23.07±12.37 |
| O (ALA30) | N-H (GLY33) | 92.66 | 3.04±0.18 | 21.80±11.90 |
| O (HIS6) | N-H (GLY9) | 88.32 | 3.08±0.18 | 28.66±9.85 |
| O (PHE10) | N-H (HIS14) | 85.24 | 2.96±0.15 | 15.74±8.93 |
| O (GLU22) | NG-HG (SER26) | 82.97 | 2.71±0.14 | 16.77±9.50 |
| OD (ASN27) | N-H (GLY29) | 79.16 | 3.08±0.18 | 38.36±11.62 |
| O (ASN27) | N-H (ILE31) | 75.10 | 3.18±0.17 | 30.36±13.27 |
| O (LYS16) | N-H (PHE20) | 74.08 | 3.13±0.18 | 23.06±12.33 |
| OD (ASN27) | N-H (ALA30) | 72.53 | 3.04±0.19 | 24.18±14.63 |
| O (VAL18) | N-H (GLU22) | 71.15 | 3.11±0.19 | 30.16±13.92 |
| O (GLN15) | N-H (PHE19) | 70.33 | 3.13±0.19 | 29.83±12.63 |
| O (ILE31) | N-H (MET35) | 70.10 | 3.07±0.18 | 21.53±11.96 |
| O (GLU11) | NE-HE (GLN15) | 69.89 | 3.00±0.18 | 23.95±10.82 |
| O (ALA21) | N-H (GLY25) | 64.71 | 3.08±0.19 | 35.49±12.83 |
| O (ASP23) | N-H (SER26) | 63.03 | 3.13±0.19 | 38.89±11.66 |
| OD (ASP7) | NH-HH (ARG13) | 53.98 | 2.85±0.13 | 23.27±11.16 |
| O (GLU22) | N-H (SER26) | 52.62 | 2.95±0.17 | 41.19±12.65 |
| O (ARG13) | N-H (LYS16) | 51.48 | 3.10±0.17 | 46.08±9.81 |
