# Supplementary material for: How Ionic Strength Affects the Conformational Behavior of Human and Rat Beta Amyloids – A Computational Study
Source: PLoS One. 2013 May 23;8(5):e62914. doi: 10.1371/journal.pone.0062914 (PMC3662769; doi:10.1371/journal.pone.0062914)
Supplement: Table S5 — Most significant intramolecular hydrogen bonds with occupancy greater than 50% of the trajectory and their geometric characteristics (donor-acceptor distance, acceptor-donor-hydrogen angles) found for amyloid with rat amino acid sequence calculated from molecular dynamics simulation for c(NaCl) = 0.15 M. (DOC) [file pone.0062914.s018.doc]

**Table S5:**

Most significant intramolecular hydrogen bonds with occupancy larger than 50 % of the trajectory and their geometric characteristics found for amyloid with rat amino acids sequence calculated from molecular dynamics simulation for c(NaCl) = 0.15 mol.dm^-3^.

| Acceptor | Donor - H | Occupancy [%] | Distance D-A [Å] | Angle A-D-H [degrees] |
| --- | --- | --- | --- | --- |
| O (HIS14) | N-H (VAL18) | 98.93 | 2.94±0.13 | 14.78±8.37 |
| O (GLU11) | N-H (GLN15) | 97.95 | 2.95±0.16 | 17.27±9.57 |
| O (LEU17) | N-H (ALA21) | 94.96 | 2.96±0.15 | 19.96±11.00 |
| O (PHE10) | N-H (HIS14) | 93.16 | 3.00±0.17 | 20.64±11.48 |
| O (ARG13) | N-H (LEU17) | 92.84 | 3.00±0.16 | 25.59±12.78 |
| O (PHE20) | N-H (VAL24) | 88.77 | 2.92±0.15 | 23.83±11.71 |
| O (PHE19) | N-H (ASP23) | 80.30 | 3.00±0.17 | 26.22±14.41 |
| O (LYS16) | N-H (PHE20) | 78.65 | 3.08±0.18 | 19.80±11.42 |
| O (SER8) | N-H (VAL12) | 77.31 | 2.87±0.14 | 14.40±7.99 |
| O (ASP7) | N-H (GLU11) | 72.69 | 3.00±0.17 | 20.23±12.28 |
| O (GLY29) | N-H (ILE32) | 65.22 | 3.14±0.18 | 23.19±12.45 |
| O (PHE19) | N-H (GLU22) | 64.99 | 3.13±0.18 | 37.67±11.87 |
| O (GLN15) | N-H (PHE19) | 64.04 | 3.17±0.18 | 31.21±13.04 |
| O (GLY25) | N-H (LYS28) | 62.91 | 3.14±0.19 | 23.99±12.06 |
| O (VAL12) | N-H (LYS16) | 61.04 | 3.16±0.18 | 21.54±12.04 |
| O (ILE41) | N-H (HIS6) | 58.15 | 3.03±0.19 | 19.70±10.48 |
| O (HIS6) | N-H (PHE10) | 54.45 | 3.17±0.18 | 35.33±13.39 |
