# Supplementary material for: How Ionic Strength Affects the Conformational Behavior of Human and Rat Beta Amyloids – A Computational Study
Source: PLoS One. 2013 May 23;8(5):e62914. doi: 10.1371/journal.pone.0062914 (PMC3662769; doi:10.1371/journal.pone.0062914)
Supplement: Table S6 — Most significant intramolecular hydrogen bonds with occupancy greater than 50% of the trajectory and their geometric characteristics (donor-acceptor distance, acceptor-donor-hydrogen angles) found for amyloid with rat amino acid sequence calculated from molecular dynamics simulation for c(NaCl) = 0.30 M. (DOC) [file pone.0062914.s019.doc]

**Table S6:**

Most significant intramolecular hydrogen bonds with occupancy larger than 50 % of the trajectory and their geometric characteristics found for amyloid with rat amino acids sequence calculated from molecular dynamics simulation for c(NaCl) = 0.30 mol.dm^-3^.

| Acceptor | Donor - H | Occupancy [%] | Distance D-A [Å] | Angle A-D-H [degrees] |
| --- | --- | --- | --- | --- |
| O (PHE20) | N-H (VAL24) | 99.54 | 2.90±0.13 | 16.45±9.58 |
| O (GLU11) | N-H (GLN15) | 98.36 | 2.92±0.14 | 19.97±11.17 |
| O (LEU17) | N-H (ALA21) | 97.88 | 2.98±0.16 | 19.25±10.26 |
| O (PHE19) | N-H (ASP23) | 89.67 | 3.03±0.18 | 26.19±13.08 |
| O (LYS16) | N-H (PHE20) | 82.86 | 3.09±0.18 | 21.18±11.62 |
| O (VAL24) | N-H (GLY29) | 78.56 | 3.08±0.18 | 27.21±13.30 |
| O (VAL18) | N-H (GLU22) | 77.00 | 3.07±0.19 | 27.60±13.52 |
| O (GLU22) | N-H (SER26) | 73.34 | 2.94±0.17 | 36.63±13.59 |
| O (PHE10) | N-H (HIS14) | 71.04 | 3.00±0.18 | 30.43±14.42 |
| O (ASP23) | N-H (ASN27) | 70.09 | 3.10±0.19 | 33.67±14.03 |
| O (ALA21) | N-H (GLY25) | 70.02 | 3.03±0.19 | 39.12±12.08 |
| O (SER8) | N-H (VAL12) | 68.81 | 2.84±0.12 | 17.00±9.62 |
| O (GLU22) | OG-HG (SER26) | 68.36 | 2.73±0.15 | 17.49±10.14 |
| O (GLU11) | N-H (LYS16) | 67.11 | 3.01±0.15 | 17.38±10.52 |
| O (GLY9) | N-H (ARG13) | 66.18 | 3.12±0.19 | 22.58±11.88 |
| O (GLY5) | N-H (GLY9) | 52.08 | 3.10±0.19 | 35.97±13.90 |
| O (HIS6) | N-H (GLY9) | 51.17 | 3.15±0.18 | 40.26±12.03 |
